# A critical role for linker DNA in higher-order folding of chromatin fibers

**DOI:** 10.1093/nar/gkab058

**Published:** 2021-02-15

**Authors:** Thomas Brouwer, Chi Pham, Artur Kaczmarczyk, Willem-Jan de Voogd, Margherita Botto, Petra Vizjak, Felix Mueller-Planitz, John van Noort

**Affiliations:** Biological and Soft Matter Physics, Huygens-Kamerlingh Onnes Laboratory, Leiden University, Niels Bohrweg 2, 2333 CA Leiden, The Netherlands; Biological and Soft Matter Physics, Huygens-Kamerlingh Onnes Laboratory, Leiden University, Niels Bohrweg 2, 2333 CA Leiden, The Netherlands; Biological and Soft Matter Physics, Huygens-Kamerlingh Onnes Laboratory, Leiden University, Niels Bohrweg 2, 2333 CA Leiden, The Netherlands; Biological and Soft Matter Physics, Huygens-Kamerlingh Onnes Laboratory, Leiden University, Niels Bohrweg 2, 2333 CA Leiden, The Netherlands; Biological and Soft Matter Physics, Huygens-Kamerlingh Onnes Laboratory, Leiden University, Niels Bohrweg 2, 2333 CA Leiden, The Netherlands; Biomedical Center, Ludwig-Maximilians-Universität München, 82152 Martinsried, Germany; Biomedical Center, Ludwig-Maximilians-Universität München, 82152 Martinsried, Germany; Institute of Physiological Chemistry, Faculty of Medicine Carl Gustav Carus, Technische Universität Dresden, Fetscherstraße 74, 01307 Dresden, Germany; Biological and Soft Matter Physics, Huygens-Kamerlingh Onnes Laboratory, Leiden University, Niels Bohrweg 2, 2333 CA Leiden, The Netherlands

## Abstract

Nucleosome-nucleosome interactions drive the folding of nucleosomal arrays into dense chromatin fibers. A better physical account of the folding of chromatin fibers is necessary to understand the role of chromatin in regulating DNA transactions. Here, we studied the unfolding pathway of regular chromatin fibers as a function of single base pair increments in linker length, using both rigid base-pair Monte Carlo simulations and single-molecule force spectroscopy. Both computational and experimental results reveal a periodic variation of the folding energies due to the limited flexibility of the linker DNA. We show that twist is more restrictive for nucleosome stacking than bend, and find the most stable stacking interactions for linker lengths of multiples of 10 bp. We analyzed nucleosomes stacking in both 1- and 2-start topologies and show that stacking preferences are determined by the length of the linker DNA. Moreover, we present evidence that the sequence of the linker DNA also modulates nucleosome stacking and that the effect of the deletion of the H4 tail depends on the linker length. Importantly, these results imply that nucleosome positioning in vivo not only affects the phasing of nucleosomes relative to DNA but also directs the higher-order structure of chromatin.

## INTRODUCTION

In eukaryotic cells, DNA is compacted into arrays of nucleosomes, forming chromatin fibers. The structure of these fibers affects the accessibility of DNA (1) and consequently plays an important role in regulating DNA transactions such as transcription, replication and repair (2–4). Nucleosome stacking interactions drive chromatin fibers into compact higher-order structures, generally referred to as 30-nm fibers. The structure of these fibers has proven difficult to resolve and have been described in terms of disordered fibers, without regular features, and 1-start (solenoid) and 2-start (zig-zag) models, in which neighboring or next-neighboring nucleosomes form one or two stacks of nucleosomes that fold in a super helix. The term 1- or 2-start refers to the stacking of nucleosomes in the folded fibers, which start with either one or two stacks. Consistent with this terminology we refer to unfolded fibers, lacking nucleosome-nucleosome interactions, as 0-start. The condensed structure of the left-handed 2-start fiber, composed of tetra-nucleosomal units, was resolved with cryo-EM (5), and was highly similar to the crystal structure of a tetra-nucleosome (6). Analogous 2-start structures have been reported based on RICC-sequencing (7) and Micro-C (8) approaches. However, both the generality of such a 2-start topology for 30-nm fibers (9–11) and their occurrence and function *in vivo* have been heavily debated (12). Whereas highly condensed, regular fibers have been reconstituted, folded and characterized *in vitro*, high-resolution EM imaging (13,14) and super-resolution optical microscopy (15) of eukaryotic nuclei rather suggest a disordered packing of nucleosomes in native chromatin fibers. Pending further high-resolution structural data, the discrepancy between these reports impedes a mechanistic understanding of chromatin folding and its role in the eukaryotic genome metabolism.

The primary structure of chromatin, i.e. the location of nucleosomes along the DNA, has been mapped with exquisite detail using nucleosome-sequencing approaches (16,17). Alignment of transcription start sites (TSS), yields decidedly regular, equally spaced nucleosome distributions near the TSS, and other barriers in the genome (18). Recently, Baldi *et al.* reported that such regular spacing of nucleosomes is more universal and is also prominent in silent promotors (19), which may have been obscured by variations in phasing of otherwise regular arrays of nucleosomes. Importantly, nucleosome spacing, quantified by the average nucleosome repeat length (NRL), varies with the functional conformation of chromatin (20). This implies that subtle, local regulation of the NRL may play an important role in genome function, possibly by modulation of the higher-order structure of chromatin.

When reporting quantized linker lengths of 10*n* + 5 bp in *Saccharomyces cerevisiae*, Widom speculated that this might be the result of constraints imposed by the higher-order structure of chromatin (21). However, the overall high transcription activity in yeast suggests an absence of condensed chromatin. Higher organisms and mature cells tend to have longer linker lengths and favor 10*n* lengths, as summarized by Perišić *et al.* (22). Though modern high-resolution genome-wide methods allow for mapping of nucleosome positions with single base pair accuracy (16), analysis of nucleosome-nucleosome interactions not only requires positional mapping of multiple nucleosomes on the same DNA substrate, it also necessitates simultaneously resolving nucleosome-nucleosome interactions. This has not been possible and structural studies on native chromatin typically average over large populations of chromatin fragments (12–15), obscuring the details of higher-order folding. The structural consequences of variations of the NRL have to date only been evaluated coarsely.

*In silico* studies of chromatin fibers, which allow a detailed energetic evaluation of single chromatin fiber structures, highlight the role of linker DNA—the DNA between adjacent nucleosomes- in the geometry and elasticity in compacting chromatin. A multitude of condensed fiber topologies has been computed for different linker lengths when using straight linker DNA and fixed nucleosome angles (23). Mesoscale modeling yielded zigzag structures for short NRL chromatin fibers and solenoid-like features for longer NRLs (22). The implications of small changes in NRL, however, seem to be best modeled by more refined rigid base pair models, that have proven successful in describing nucleosome positioning (24). By fixing nucleosome orientations in a 2-start topology, Norouzi and Zhurkin reported a 10 bp periodicity in fiber compaction (25–27) and quantified the energetic penalty for non-optimal NRLs.

To experimentally resolve the role of NRL in chromatin higher-order structure, tandem arrays of the Widom 601 nucleosome positioning sequence have been instrumental (28). Though any NRL can be cloned into a regular array (29) and reconstituted with nucleosomes, experimental work has almost exclusively focused on 10*n* NRLs, yielding high-resolution X-ray structures of 157 (30) and 167 (6) NRL fibers, single-particle cryo-EM reconstructed structures of 177 and 187 NRL fibers (5), as well as quantification of the fiber diameter of 177–237 NRL fibers in 10 bp steps by EM (9). It is likely that this focus on 10*n* NRLs not only reflects earlier reports on NRLs found *in vivo*. It may also be that the better folding properties of these fibers make it easier to handle them experimentally, but little experimental data are available for intermediate NRLs.

Our previous force spectroscopy studies, which uniquely probe single chromatin fibers without staining, fixation or surface deposition, showed that unfolding of 167 NRL fibers under physiological conditions could best be interpreted by 2-start structures, whereas unfolding of 197 NRL fibers could better be described with a 1-start unfolding model (10,11,31). Many of these 10*n* NRLs have also been scrutinized using other biochemical and biophysical techniques, such as sedimentation analysis, atomic force microscopy (AFM) and single-molecule force spectroscopy. Most notably, Grigoryev *et al.* used sedimentation analysis, AFM and EM to quantify chromatin compaction as a function of small NRL increments (32). A 10 bp periodic dependence of chromatin folding for NRLs in the range of 165–177 bp was found and the highest level of condensation was found for 20 and 30 bp linker DNA. However, a systematic experimental analysis of chromatin fiber structure with single base pair increments of the NRL is lacking.

Here, we combine rigid base pair Monte Carlo (rbMC) simulations with single-molecule force spectroscopy to probe a series of chromatin fibers and uncover the detailed implications of NRL changes on fiber folding with single base pair increments. The rbMC simulations allow detailed evaluation of chromatin structures with different topologies and quantitative comparison of the energetic cost of linker DNA distortions that nucleosome stacking imposes. Previously, we have shown that rbMC simulations, in which histone–DNA and histone–histone interactions are implemented by simple harmonic potentials, can reproduce experimental force–extension curves of folded fibers fairly well (33). In this study, we used magnetic tweezers to measure the mechanical properties of a large set of reconstituted fibers and combined the results with the insight obtained from simulations. Furthermore, we quantified nucleosome-nucleosome stacking energies and investigated how much the H4 tail contributes to nucleosome stacking in short and long NRL fibers. Previous studies showed that A-tracts have a profound impact on inter-nucleosomal orientation and higher order chromatin folding, raising the question whether other linker sequences also affect fiber folding (34). We therefore tested whether the sequence of the linker DNA could affect the mechanical properties of chromatin (35). Overall, we find that the mechanical constraints imposed by the linker DNA define if and with which other nucleosomes the nucleosome stacks, and thereby direct nucleosomal arrays into specific higher-order structures.

## MATERIALS AND METHODS

### rbMC simulations

**Figure 1.**
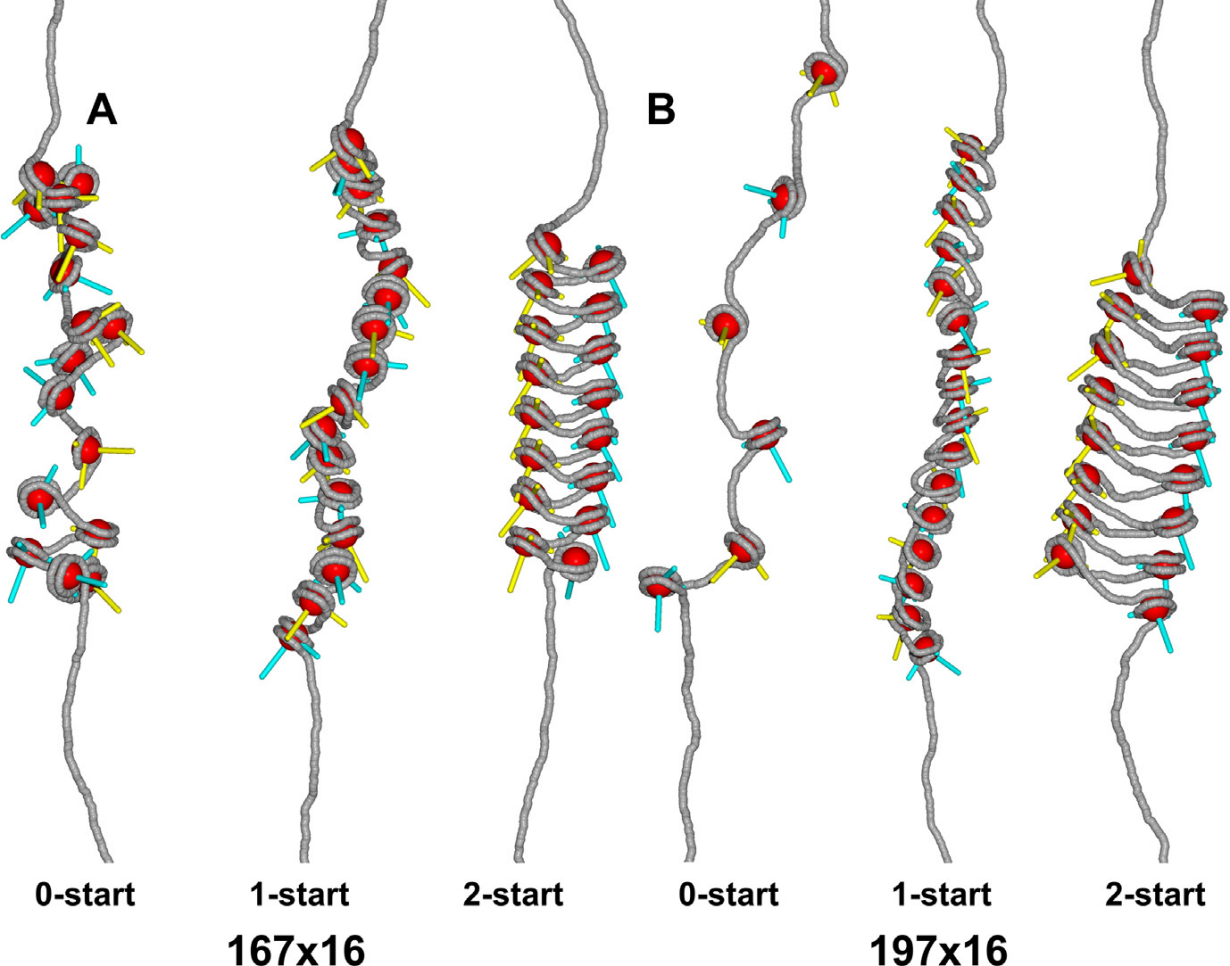
Nucleosome stacking defines the higher-order structure of chromatin fibers. The spatial orientation of linker DNA (grey) and nucleosomes (red) is depicted in three chromatin fiber structures: 0-start, 1-start and 2-start. In this simulation, 3 pN of force was applied to stretch the fiber structures (33). Nucleosome orientation was indicated by the blue and yellow rods, pointing to the dyad of the nucleosome and the direction along the superhelical axis. (**A**) The relatively short linker DNA in NRL 167 fibers constrains nucleosome stacking in chromatin fibers. The 1-start fiber required strong linker DNA bending to accommodate nucleosome stacking. The linker DNA in the 2-start fiber was less bent. (**B**) NRL 197 fibers can more easily facilitate the linker bending imposed by 1-start fibers. The linker DNA of the 2-start fiber featured only moderate bends.

**Figure 2.**
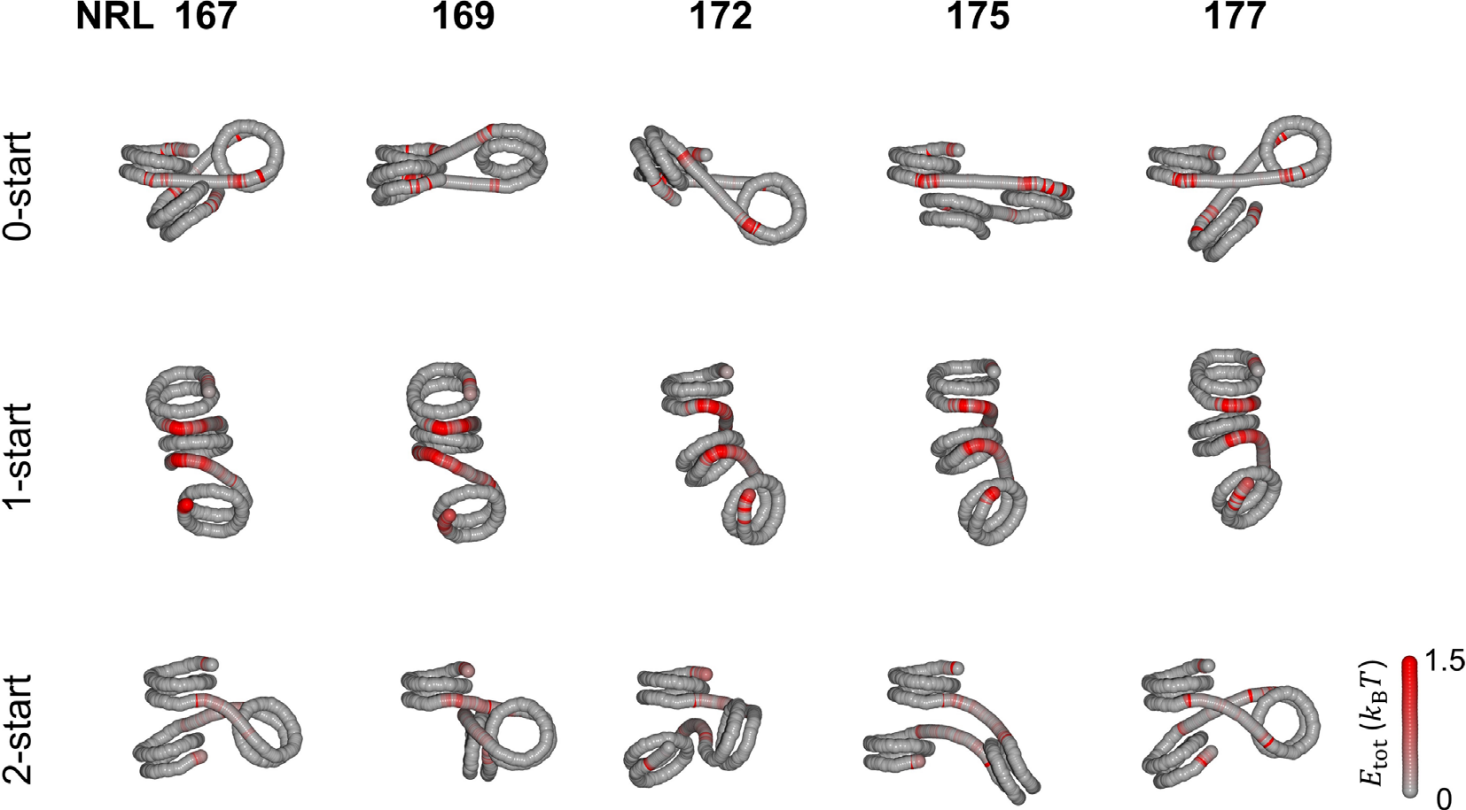
Bending of short (20–30 bp) linker DNA imposes large energy penalties for 1-start fibers and compromises stacking in 2-start fibers. The average conformation of the DNA of three adjacent nucleosomes, including their linker DNA, was calculated for the 0-start, 1-start and 2-start structures. The tension in the individual base pairs in the linker DNA is represented by the red color, which shows the bending energy compared to the starting, minimal energy structure. When no stacking interactions were simulated, the linker DNA was on average straight. In this conformation, nucleosome breathing was observed at the outer contact points of each nucleosome, resulting in red base pairs that reflect an increased standard deviation of their coordinates as compared to the start conformation. The bending of the linker DNA in a 1-start structure required strong deformation of the linker DNA, indicated by large energy penalties. NRL 167 fibers showed approximately straight linker DNA in a 2-start structure. Increasing the linker length imposed deviations from optimal stacking, which was maximal for NRL 172. Increasing the linker length to NRL 177 recovered proper stacking.

**Figure 3.**
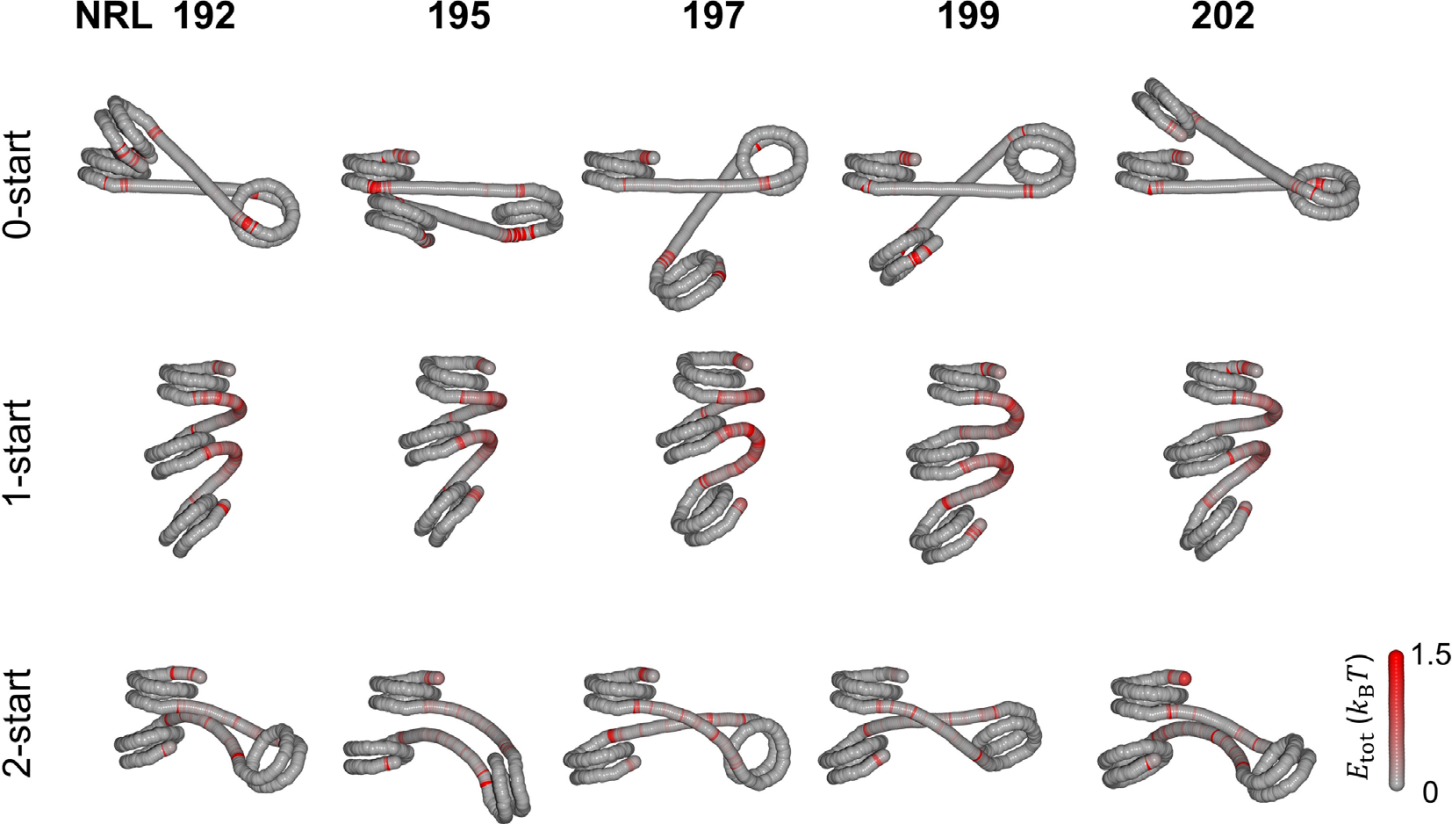
Longer (45–55 bp) linker lengths can better accommodate linker DNA stress in 1-start fibers. 0-start structures with straight DNA featured nucleosome breathing. The increased linker DNA significantly reduced the stress in linker DNA imposed by the 1-start stacking as compared to 167–177 NRL fibers. A large variety of nucleosome orientations was found in 2-start structures in which linker DNA was slightly deformed.

**Figure 4.**
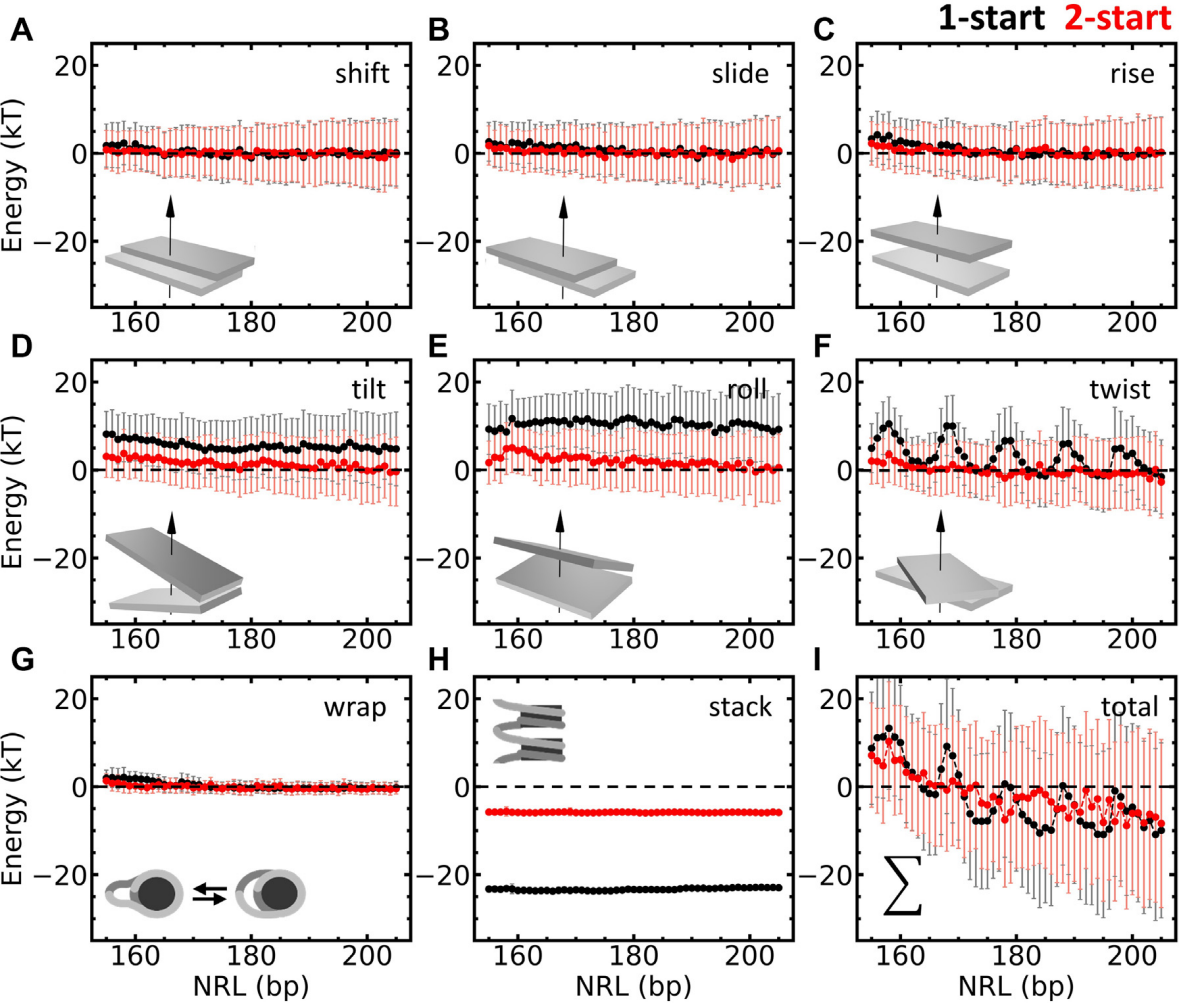
Rigid base pair Monte Carlo simulations decompose the energy contributions of fiber folding into linker DNA deformation, DNA unwrapping, and nucleosome stacking. rbMC simulations were used to quantify the total energy penalty for each base pair step associated with nucleosome stacking. 1-start fibers are depicted in black, 2-start fibers in red. All energies are relative to the energies computed for 0-start fibers with the same linker length. The energy contributions relative to the 0-start fiber were split in shift (**A**), slide (**B**), rise (**C**), tilt (**D**), roll (**E**), twist (**F**) and nucleosome wrapping (**G**) and stacking (**H**) parameters. Increasing NRLs resulted in decreased the energies of most degrees of freedom (DoF). The exception was the twist energy (f) of 1-start fibers, which was strongly modulated by NRL and oscillated with a period of 10 bp and an amplitude of approximately 10 k_B_T. The energies of rotational degrees of DoF of the 1-start fiber were higher than those of the 2-start fiber. The 2-start fiber however, yielded a much larger stacking energy than the 1-start fiber. (**I**) When all contributions are summed, the negative energy difference with the 0-start fiber for NRLs larger than 165 bp indicates that nucleosome stacking is favored. The summed energy also exhibits a periodic average modulation of the energy with NRL for 1-start fibers, but differences between 1- and 2-start folding are smaller than the standard deviation of the simulated energies.

Chromatin fibers were simulated using a rigid base pair model, expanded to include (un)wrapping of nucleosomal DNA and (un)stacking of nucleosomes in one-start and two-start chromatin fibers (33). Chromatin fibers under force, as shown in Figure 1, were simulated following the approach by De Jong *et al.* (33). Fibers with 16 repeats of NRL 167 and NRL 197 were simulated without nucleosome stacking (0-start), stacked in a 1-start structure, or stacked in a 2-start structure. Stacking energy was set at 25 ${k_{\rm{B}}}T,$ and wrapping energy at 2.5 ${k_{\rm{B}}}T$ per contact point. Note that we did not include a force in the simulations presented in Figures 2–4, which resulted in stacked nucleosomes throughout all simulations, except for the 0-start conformation. More details can be found in supplementary information.

### DNA substrates

The DNA substrates, containing an array of 601 nucleosome positioning sequences (28), were constructed as described by Wu *et al.* (29). An insert containing a single nucleosome positioning sequence was cloned between the XbaI and EcoRI site in a pUC18 plasmid. The plasmid was split in two fractions: an acceptor and a donor plasmid. The acceptor plasmid was digested with BstXI and DraIII and purified. The donor plasmid was digested with BstXI and BglI, which produced a backbone and an insert containing the desired sequence. We introduced phosphatase to prevent self-ligation. The insert was purified and ligated into the acceptor plasmid by T4 ligase. The ligated plasmid then contained two identical inserts. The plasmid was subsequently transformed into *Escherichia coli* DM1-blue competent cells, amplified and harvested. Repeating these steps doubled the number of inserts in the plasmid, which was done three times to create arrays of 16 inserts. Sequences are listed in supplementary information.

### Chromatin reconstitution

Plasmids were linearized with BsaI and BseYI, creating linear arrays with $\sim$1000 bp of DNA handles on each side. A digoxigenin was introduced at the BsaI site and a biotin at the BseYI site with a Klenow reaction (36). Chromatin was reconstituted following (37). DNA and human WT histone octamers (Epicypher, Durham, USA) were added together in 2M sodium buffer (2 M NaCl/1× TE buffer), which was slowly dialyzed to 200 mM NaCl. The level of saturation of the chromatin fibers depended on the ratio between 601 nucleosome positioning sequences and histone octamers: excess histones yielded additional tetrasomes and nucleosomes on the DNA handles. A too low stoichiometry resulted in undersaturated fibers. More details and a step-by-step protocol for DNA labeling and chromatin reconstitution can be found in (38). Drosophila WT and gH4 histones were purified, reconstituted into histone octamers and purified as described (39).

### Force spectroscopy on single chromatin fibers

All force spectroscopy experiments were done on a home-built microscope. Details can be found in supplementary information. The force–extension curves were measured by increasing the force to 9 pN, releasing the force to 0.5 pN, and increasing the force to 55 pN, and releasing again. This trajectory was chosen to minimize the sticking of the beads to the cover slip. Usually, a small force was sufficient to unstick a bead. A force of 0.5 pN was enough to keep the bead suspended above the cover slip whilst still fully compacting the fiber. The magnets were moved at a constant speed of 0.25 mm per second, which increased or decreased the force exponentially.

### Statistical mechanics model

To infer mechanical properties of individual chromatin fibers the force–extension curves were fitted to a statistical mechanics model developed by Meng *et al.* (31). All curves that contained more than 12 nucleosomes, as assessed by the number of 25 nm steps at *F* > 10 pN, were included in further analysis. The model and fitting procedure are described in supplementary information.

## RESULTS

### rbMC simulations yield the detailed conformation of linker DNA

To quantify the geometrical constraints on nucleosome stacking in folded chromatin fibers, we performed a set of rbMC simulations in which we evaluated three structures: a fiber devoid of nucleosome-nucleosome interactions (0-start), a solenoid fiber (1-start), and a zig-zag fiber (2-start). Nucleosome stacking was implemented by nucleosome step parameters in 6 degrees-of-freedom (DoF), analogous to base pair step parameters (33). Figure 1 shows snapshots of states of NRL 167 and NRL 197 fibers. The fibers were gently stretched by 3 pN force to provide an unobstructed view on fiber folding. As expected, and particularly visible in Figure 1B, the 0-start structures were most elongated and featured some unwrapping of the nucleosomal DNA. Nucleosome stacking largely inhibited such unwrapping in 1-start and 2-start fibers. As reported before (33), the linker DNA was more bent in 1-start than in 2-start structures, but nucleosome stacking was better accommodated in 1-start fibers. The harmonic stacking and wrapping potentials may require further refinement to properly discriminate between the different topologies. Nevertheless, the rbMC simulations allow for a detailed, qualitative comparison of the linker DNA in different structures and are well suited to evaluate changes upon increasing the linker length.

In Figures 2 and 3, we show the changes in the conformation of the linker DNA for the three topologies as the linker length is increased in small steps. The structures were reconstructed from the average step parameters of the DNA of the first three nucleosomes after 10 000 Monte Carlo iterations at zero force. To pinpoint large deformations of the linker DNA, the deviations in the step parameters from unperturbed DNA were converted to energies per base pair and depicted in red (see Materials and Methods). In the absence of stacking, the linker DNA was approximately straight. Excluded volume effects, incorporated in our simulations, did not impose enough constrains to yield large deformations of the linker DNA in any of the linker lengths of the 0-start fiber. The base pairs depicted in red near the end of the nucleosomes reflect unwrapping of the nucleosomal DNA. As the linker length increases, the orientation of adjacent nucleosomes rotates, as every additional base pair twists the adjacent nucleosome by ∼36°.

When neighboring nucleosomes were forced to stack into a 1-start fiber, bending of the linker DNA imposed large energy penalties, depicted in Figure 2 by the bright red color of the linker DNA. Nucleosome pairs were positioned in very similar orientations, showing that stacking in the 1-start structure could be achieved for all linker lengths. Little difference in terms of structure and energy penalties was observed as the linker length increased from 20 to 30 bp. Perhaps surprisingly, there was little unwrapping of the nucleosomal DNA in the 1-start fibers, except for the nucleosomal DNA in the first half of the bottom nucleosome, which was not constrained by stacking.

When nucleosomes were forced into a 2-start fiber, the linker DNA was straighter, yielding lower energy penalties. Like in the 1-start state, nucleosome unwrapping was repressed in the 2-start fiber. The relative orientation of the first and the third nucleosomes, however, varied strongly as the linker DNA length increased from 20 to 30 bp. Especially the NRL 169, NRL 172 and NRL 175 structures deviated considerably from the optimal stacking geometry. Apparently, nucleosome stacking was more restricted by the linker DNA in 2-start fibers than in 1-start fibers, resulting in a large variety of higher-order structures. Chromatin fibers with NRL 167 and NRL 177 had the straightest linker DNA and appeared better stacked. In summary, the rbMC simulations of fibers with 20–30 bp of linker DNA shown in Figure 2 featured different geometries in which the 1-start fibers featured most stress in the linker DNA, while 2-start fibers compromised stacking to reduce linker DNA stress.

For linker lengths between 45 and 55 bp, we observed similar trends, as shown in Figure 3. The energy penalties for chromatin with longer linker DNA were, however, considerably lower since stress was distributed over more base pairs. In the absence of nucleosome stacking, chromatin fibers with longer linkers sustained straight DNA and nucleosome breathing. In 1-start fibers, the stress appeared to accumulate at distinct locations that were spaced by 10 bp, but nucleosomes positions and orientations were quite similar in the series of simulations. The linker DNA of 2-start fibers was only gradually bent and the stress appeared to be well distributed along the linker DNA. The nucleosomes appeared similarly stacked, independent of the linker length although the relative orientation of the two staked nucleosomes varied a lot, which may have a large impact on the resulting higher-order structures.

### Decomposition of energy distributions as a function of NRL highlights twist as a decisive factor in fiber folding

To quantify the effect of linker length in more detail, we calculated the average folding energy per nucleosome. Each energy contribution, relative to the 0-start fiber structure, was calculated by summing the energy terms over all base pairs between two dyads. In Figure 4, the contributions of each degree of freedom are plotted as a function of NRL. Overall, the energy contributions of the linker DNA decreased with linker length, as stacking could be better accommodated when the resulting stress is distributed over longer linker DNA. The translational step parameters (shift, slide, rise) showed little difference between the 1-start and 2-start states. The deformation of the linker DNA was more prominent in the rotational step parameters (tilt, roll, twist): folding fibers into a 1-start conformation costs up to 8 k_B_T for tilt and 10 k_B_T for roll. The stress in the linker DNA in 2-start fibers was significantly less, starting at a few k_B_T for tilt and roll in NRL 167 and approaching zero for longer NRLs. The two states differ most in terms of twist: whereas 2-start fibers featured a small but gradual decay in twist energy as the linker length increases, 1-start fibers displayed a 10 bp periodic modulation of the twist energy. The most favorable linker lengths at 10*n* base pairs were up to 10 k_B_T lower in energy than the most unfavorable ones.

Next to the energy contributions due to changes in the base pair steps, we plotted the changes in wrapping and stacking energy in Figure 4. Unwrapping of nucleosomal DNA contributed only marginally to energy differences between the three structures. The outcomes of the rbMC simulations at low force, as reported here, did not depend on the precise value that was chosen for the stacking energy, provided that it was large enough to impose stacking. However, when compared with non-stacked nucleosomes, as done in Figure 4h and i, the difference in free energy shifted these curves up when smaller values for the stacking energy were chosen. Nucleosome stacking lowered the energy per nucleosome by approximately 23 k_B_T in 1-start fibers, which is about 2 k_B_T less than the maximum stacking energy, showing that the simulations resulted in a compromise between optimal stacking and minimization of the stress in the linker DNA. For 2-start fibers, the less perfect stacking only lowered the energy by 6 k_B_T as compared to 0-start fibers. It therefore seems that 2-start fibers prefer to compromise stacking when the linker length is not optimal, whereas 1-start fibers rather accommodate stacking by twisting the linker DNA.

In summary, the total energy per nucleosome pair was dominated by contributions of the rotational step parameters. The 0-start state appeared to have the lowest energy for very short NRLs. Beyond approximately 165 bp the minimum energy alternated between the 2-start and the 1-start state. For NRLs that have an optimal twist, 1-state conformations appear to be preferred. Note however, that the standard deviations that were computed exceeded the difference in energy between the two topologies, suggesting that all three conformations can be sampled, given enough time. Though the trends that we observed are clear and highlight torsional stress rather than bending of linker DNA as the defining aspect of fiber folding, we want to emphasize that the harmonic approximation of the stacking energy may not be sufficient for absolute quantitative comparison (see Discussion). Nevertheless, based on these simulations we expect a strong dependence of fiber folding on the linker length, featuring a 10 bp periodicity of its stability.

### Single-molecule force spectroscopy reveals a 10 bp periodic dependence of fiber folding parameters on NRL

To experimentally examine the role of linker DNA on chromatin fiber structure in a systematic manner, we performed a series of force spectroscopy experiments on chromatin fibers with increasing NRLs. A series of 22 tandem arrays of 16 601 positioning sequences were cloned using the method of Wu *et al.* (29) (see Materials and Methods). The linker length was varied in single base pair increments in range NRL 167–177 (*short* linker DNA) and NRL 192–202 (*long* linker DNA). Both series cover one helical period of the DNA, which should result in a full turn of each nucleosome with respect to its neighbor in 0-start fibers.

**Figure 5.**
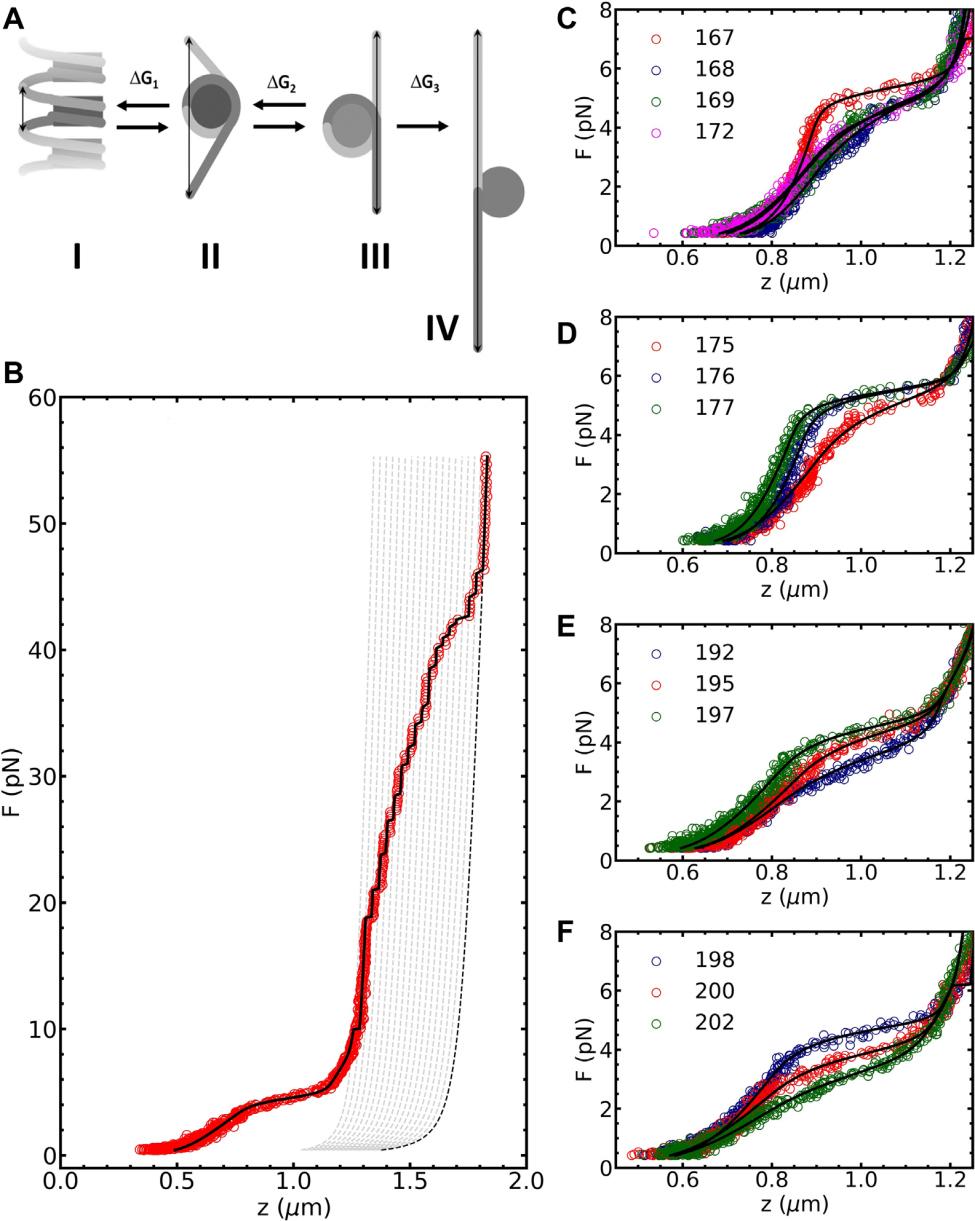
Force spectroscopy experiments show that single base pair changes in linker length have large effects on fiber folding. (**A**) chromatin fibers unfold in three steps and switches between four conformations: I) Folded fibers consist of stacked nucleosomes. II) Unstacking yields a partially unwrapped nucleosome and costs rupture energy $\Delta {G_1}$. III) Subsequent unfolding yields singly wrapped nucleosomes and costs unwrapping energy $\Delta {G_2}$. IV) At high force, the DNA is completely unwrapped from the histone core at the cost of unwrapping energy $\Delta {G_3}.$ (**B**) Fitting a typical force–extension curve of a chromatin fiber (NRL 197) with the statistical mechanics model yielded 18 stacked nucleosomes, fiber stiffness $k$ = 0.3 ± 0.1 pN/nm, rupture energy $\Delta \ {G_1}$= 23 ±1 k_B_T, and partial interaction energy $\Delta {G_2}$ = 8 ±1 k_B_T. (**C**) A qualitative comparison of chromatin fibers with NRL 167, NRL 168, NRL 169 and NRL 172 revealed that fibers with NLR 167 unfolded cooperatively and had the highest $k$ and $\Delta {G_1}$. Increasing the linker length by a single base pair to NRL 168 distorted fiber folding, reflected by the loss of cooperativity and the sharp drop in $k$ and $\Delta {G_1}$. Increasing the linker length to NRL 169 or NRL 172 did not have a subsequent effect. $\Delta {G_2}$ was the same for all NRL. (**D**) For almost 10 bp longer linker length the reverse trend was observed. NRL 175 depicted gradual unfolding, low stiffness and reduced rupture force. Increasing the linker length to NRL 176 restored the shape of the curve to the one observed for NRL 167. Further increase of the linker length to NRL 177 had no effect. Again, $\Delta {G_2}$ remained unaffected by NRL. (**E**) A different trend was observed for longer linker DNA. Chromatin fibers with NRL 192 had a relatively low $k$ and $\Delta {G_1}$, which steadily increased for NRL 195 and NRL 197. (**F**) NRL 198, NRL 200 and NRL 202 showed a decline in $k$ and $\Delta {G_1}$. The fitted parameters of all curves in c-f) can be found in Table S1 of the supplementary material.

Figure 5A schematically shows the unfolding of a nucleosome that is embedded in a folded fiber, in which several characteristic force-induced intermediate conformations occur (31). A typical experimental force–extension curve of a chromatin fiber is shown in Figure 5B. At forces below 3–5 pN, the nucleosomes are predominantly stacked in a folded fiber (conformation I in Figure 5A shows a 1-start fiber, but the same statistical mechanics model, with adjusted parameters can account for 2-start fibers). When the force increases, the nucleosomes unstack into a partially unwrapped conformation (conformation II). Further unwrapping yields a singly wrapped conformation in which 77 bp are wrapped around the histone core (conformation III). At forces above 10 pN, the singly wrapped nucleosomes unfold into a fully unwrapped conformation that has an extension that equals that of bare DNA (conformation IV). This last transition is not in equilibrium, as opposed to the first two transitions, and results in distinct steps of approximately 25 nm for each nucleosome (31).

Four exemplary force–extension curves of chromatin fibers with NRL 167, 168, 169 and 172 are plotted in Figure 5C. Compared to the NRL 167 fiber, the NRL 168, 169 and 172 fibers were easier to stretch and ruptured at smaller forces. The larger stiffness and rupture force of the NRL 167 fiber were recovered when the NRL approached 177 bp (Figure 5D). For the longer NRLs, we obtained a similar, but more gradual trend (Figure 5E and F). For both short and long linkers, linker lengths near 10*n* bp were stiffer and ruptured at larger forces than intermediate NRLs. Thus, single base pair increments of the NRL had a large impact on the fiber's mechanical response to force.

The mechanical properties of each of the four unfolding conformations, as well as the equilibrium transitions between them, were fitted to a statistical mechanics model that is based on the mechanical properties of DNA, the strength of nucleosome-nucleosome interactions, the histone–DNA interactions, and the composition of the chromatin fiber (31). Details are described in Materials and Methods. Here, we focus on the parameters that describe the rupture of the folded chromatin fiber. For all measurements, we found that the experimentally obtained force–extension curves in the force regime below 3 pN could be described by the combination of a worm-like chain (WLC) for the free DNA handles, and a Hookean spring with stiffness $k$ for the chromatinized part of the tether. The limited force range and the relatively small extension over which the fiber is stable may explain the success of this linear approximation.

Because we found that fibers reconstituted with tandem arrays of 601 sequences still featured heterogeneity in terms of the number of reconstituted nucleosomes, we manually counted the number of 25 nm steps at $f >10$ pN and attributed these to the number of nucleosomes. A typical result is depicted by the grey dotted lines in Figure 5B. Between fibers, the number of nucleosomes varied between 12 and 20, but was typically centered around 16 for 16 repeats of the 601 positioning sequence. The highly reproducible value of the second rupturing energy $\Delta {G_2}$ for all NRLs implies that this individual assessment of the composition of each fiber worked well. When comparing fibers with identical linker sequence but a different number of repeats (both NRL 167 and NRL 197), the distribution of mechanical fitting parameters overlapped (see, for example, Figure 8), which implies that the variation in the composition of individual chromatin fibers did not affect the fit results.

For different NRL fibers, the obtained fitting parameters, i.e. stiffness $k$, rupture energy $\Delta {G_1}$, and partial unwrapping energy $\Delta {G_2},$ showed a large variation that should be attributed to differences in fiber folding. Fitting the curves in Figure 5C–F yielded the highest stiffness $k$ = 1.1 ± 0.1 pN/nm and rupture energy $\Delta {G_1}$ = 23 ± 1 k_B_T for NRL 167, in agreement with our qualitative assessment. For NRL 168, 169 and 172, both the stiffness and the rupture energy dropped, which was ultimately recovered for NRL 176 bp. The chromatin fibers with longer linker lengths had a 2–3 times smaller stiffness than that of the short NRLs. The rupture energy $\Delta {G_1}$ varied between 15 and 25 k_B_T.$\ \Delta {G_2}$ was unaffected by NRL, as would be expected since at this stage the fibers are stretched far enough to prevent interactions between nucleosomes. The difference in contour length between the partially wrapped and the singly wrapped nucleosomes was fixed at 15 bp, which would correspond to two histone–DNA contacts of each about 3.5 k_B_T, in fair agreement with previous estimations (10,40). For the intermediate NRLs we obtained good fits and parameters that are consistent with these trends. Thus, all effects of changing the NRL could be captured in two fit parameters: the fiber stiffness and the nucleosome-nucleosome stacking energy. Representative examples of experimental curves for all NRLs and their fits are plotted in Supplementary Figure S1. The fitted parameters and standard errors are given in Table S5 of the supplementary material.

**Figure 6.**
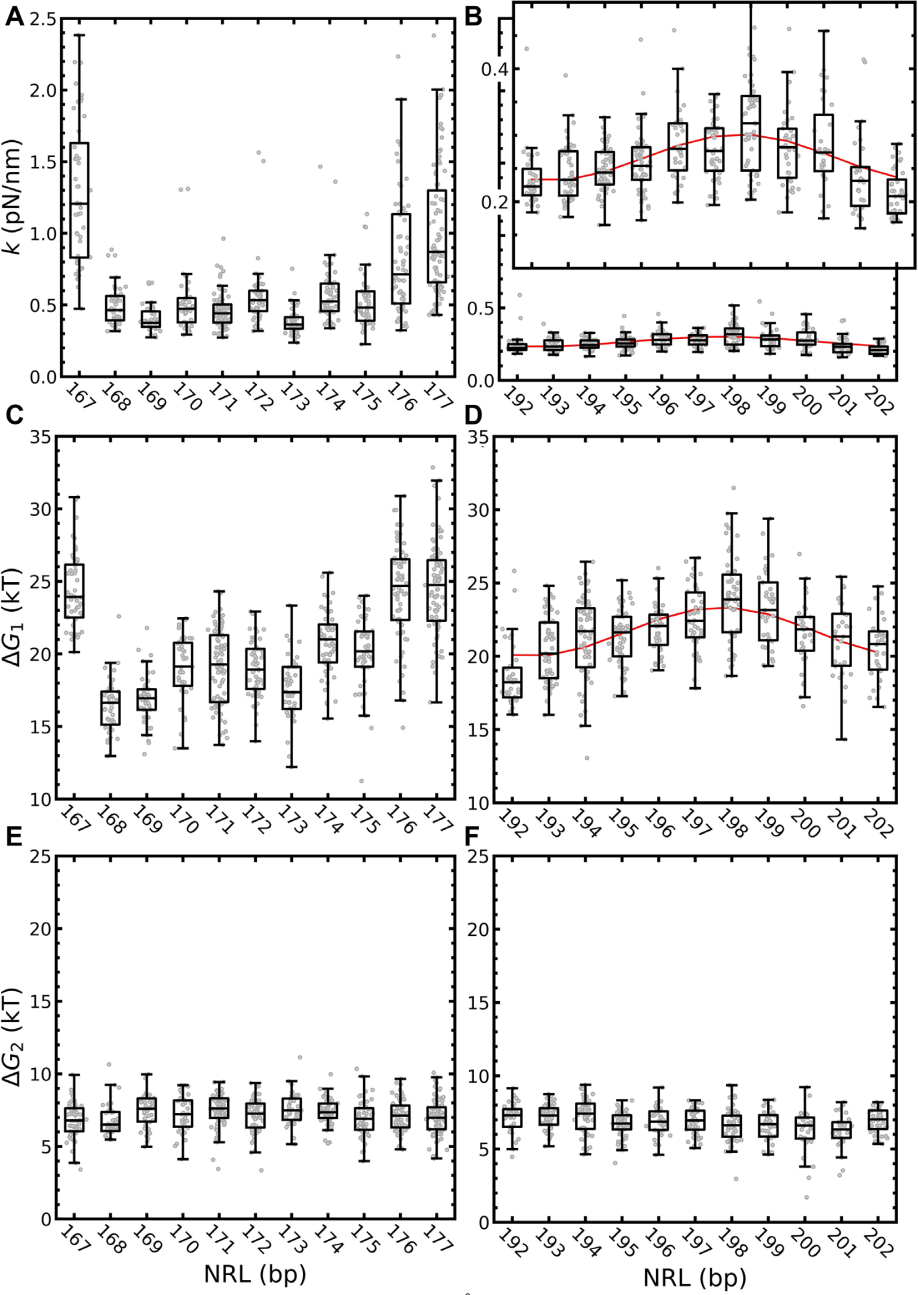
Base pair step parameters feature a 10 bp NRL periodicity. (**A**) For short NRLs, $k$ peaked at NRL 167, NRL 176 and NRL 177. Strikingly, the stiffness dropped sharply between NRL 168 and NRL 175, illustrating the sensitivity of $k$ for linker DNA length. (**B**) For long NRLs, $k$ described a gradual modulation which could be captured by a sinusoid, peaking at NRL 198 ($k\ = \ 0.3 \pm 0.1$ pN/nm). (**C**) $\Delta {G_1}$ showed a similar trend as $k$; for short linker lengths peak values were found for NRL 167, NRL 176, and NRL 177 and dropped in between. (**D**) Also for long linker lengths $\Delta {G_1}$ oscillated with a period of 10.4 bp and peaked at NRL 198, similar to $k$. (**E, F**) As expected for non-interacting nucleosomes, $\Delta {G_2}$ did not change with NRL.

To quantify variations between individual fibers from the same batch, we measured and fitted the force–extension curves of at least 35 different fibers for each of the 22 batches of different NRL that we reconstituted. The resulting fit parameter distributions are plotted as decorated box plots in Figure 6. Next to the trends described above, we observed a qualitative difference between the short and the long NRL series. Whereas the long NRLs show a gradual periodic modulation of the average stiffness and rupture energy $\Delta {G_1}$, that we fitted with a sinusoidal function with a period of 10.4 bp, the short NRL series featured a much more abrupt drop (NRL 167 to NRL 168) and recovery (NRL 175 to NRL 176) of $k$ and $\Delta {G_1}$. Apparently, an increase of 1 bp in NRL has a larger effect on short NRLs than on long NRLs. We tentatively attribute the abruptness of the transition and the relatively small values of both stiffness $k$ transition and rupture energies $\Delta {G_1}$ in between to inhibition of nucleosome stacking. The rupture energy $\Delta {G_1}$ would, in absence of stacking interactions, correspond to the partial unwrapping of nucleosomal DNA from non-interacting nucleosomes rather than unstacking. Interactions between histone tails and the (linker-) DNA could add to the stability of such non-interacting nucleosomes. Independent of the interpretation of the fit parameters, these single-molecule force spectroscopy experiments and subsequent analysis clearly reveal a 10 bp periodic modulation of fiber folding with NRL, with a larger impact for short NRLs than for long NRLs.

### H4 tails are essential for 2-start fiber folding, but not for 1-start fiber folding

**Figure 7.**
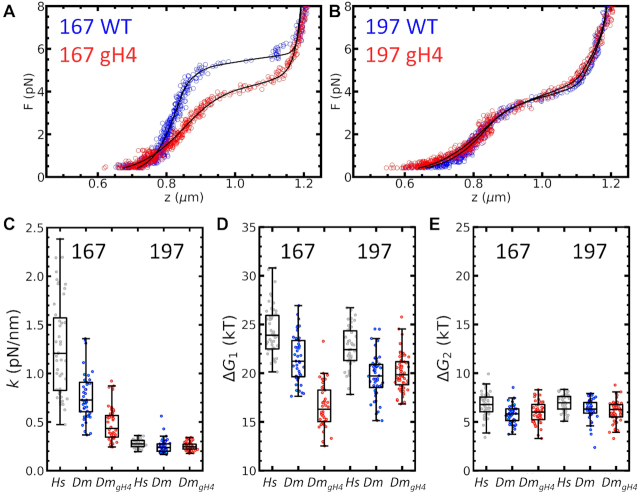
Deletion of the H4 tail affects folding in NRL 167, but not in NRL 197 chromatin fibers. (**A**) 167 NRL Fibers reconstituted with *Dm* WT histones (blue dots) unfolded similarly as those with *Hs* WT histones. Chromatin fibers reconstituted with *Dm* gH4 mutant histones (red dots) unfolded at a lower force. (**B**) Surprisingly, deletion of the H4 tail had no observed effect on the mechanical properties of NRL 197 fibers. Fit parameters (black lines) are given in Table S3 of the supplementary material. Irrespective of NRL, *Hs* WT chromatin fibers had a slightly higher $k$, $\Delta {G_1},$ and $\Delta {G_2}$ than *Dm* WT fibers. (**C**) In NRL 167 fibers, *Dm* gH4 reduced the fiber stiffness $k$ significantly compared to WT histones (*P* < 0.001), while NRL 197 fibers were unaffected (*P* = 0.98). (**D**) Unstacking energy $\Delta {G_1}$ showed the same trend: *Hs* WT histones featured a slightly higher $\Delta {G_1}$ than *Dm* WT histones, both for NRL 167 and NRL 197. Deletion of the H4 tail reduced $\Delta {G_1}$ for NRL 167 (*P* < 0.001). For NRL 197 the unstacking energy was not different (*P* < 0.35). The much lower $\Delta {G_1}$ for *Dm* gH4 chromatin with NRL 167 suggests an absence of higher-order structure. (**E**) As expected, the partial unwrapping energy $\Delta {G_2}$ remained constant irrespective of NRL or the presence of the H4 tail (*P* = 0.43 for NRL 167, *P* = 0.41 for NRL 197). The number of experiments per fiber type was 47 ± 6 (mean ± std). The average and standard deviations of the fitted parameters are given in Table S4 of the supplementary material.

To obtain further insight into the balance between nucleosome stacking and linker DNA deformation, we tested the effect of deleting the N-terminal tail of histone H4, resulting in so called globular H4 (gH4). Deletion of this tail would reduce the stacking between nucleosomes in folded fibers. We reconstituted chromatin fibers with recombinant *Drosophila melanogaster* (*Dm*) histones and compared the fiber unfolding characteristics of fibers reconstituted with both wild-type (WT) and gH4 histone with those reconstituted with human (*Hs*) histones, as used in the previous paragraphs. As shown in Figure 7A and B, the force–extension curves for both NRL 167 and NRL 197 fibers featured the characteristic unfolding plateau at 3–5 pN, and all curves fitted well to our unfolding model. Fibers reconstituted with *Dm* WT histones yielded a smaller $k$ and a lower $\Delta {G_1}$ than WT *Hs* histones. The second rupture energy, $\Delta {G_2}$, was only marginally reduced. Thus, chromatin fibers containing WT *Hs* nucleosomes folded slightly better than fibers reconstituted with WT *Dm* histones.

Deletion of the H4 tail impeded folding of NRL 167 fibers, as shown in Figure 7A. The fibers unfolded more gradually and at a lower force than with *Dm* WT histones. Fitting the model yielded a lower $k$ and $\Delta {G_1}$: $k$ dropped from $0.8 \pm 0.4$ to $0.5 \pm 0.2$ pN/nm (*P* < 0.001), and $\Delta {G_1}$ dropped from $21 \pm 2$ to $17 \pm 2$ k_B_T (*P* < 0.001) for *Dm* versus *Dm*_gH4_ nucleosomes in 167 NRL fibers. Both the stiffness and the rupture energy of the first transition had values that were similar to those of NRL 168 fibers, for which we attributed the reduced values to inhibited nucleosome stacking. In addition, the change from cooperative to uncooperative rupture of the fiber, as apparent from the more gradual rupturing plateau, is characteristic for the absence of nucleosome stacking in a 2-start structure. As expected, $\Delta \ {G_2} = \ 6 \pm 1$ K_B_T was not affected by H4 tail deletion (*P* = 0.43). These results indicate that nucleosome stacking in NRL 167 fibers was primarily mediated by the H4 tail, and support the interpretation that short non-10*n* linker chromatin fibers are best described by a 0-start state, in which nucleosomes do not stack.

Surprisingly, folding of NRL 197 fibers was unaffected by H4 tail deletion, as shown by the overlapping curves in Figure 7B. Both WT and gH4 fibers unfolded gradually at approximately 3 pN. Fitting the model to gH4 fibers yielded $k\ = \ 0.3 \pm 0.1$ pN/nm and $\Delta \ {G_1} = \ 20 \pm 2$ k_B_T. These values were the same for WT H4 histones (*P* = 0.98 and *P* = 0.35) for *Dm* versus *Dm*_gH4_ nucleosomes in 197 NRL fibers. $\Delta {G_2}$ was also unaffected by H4 tail deletion (*P* = 0.41). Note that in fibers reconstituted with gH4, $\Delta {G_1}$ was larger in NRL 197 fibers than in NRL 167, indicating that despite the lack of the H4 tail, other nucleosome-nucleosome interactions contributed to an increased rupture energy. As opposed to the NRL 167 fibers, the stiffness was unaffected, supporting the interpretation that additional contacts mediate the stacking of nucleosomes in NRL 197 chromatin fibers. Thus, 197 NRL fibers are organized differently than 167 NRL fibers, and 197 NRL fibers reach similar compaction when H4 tails are deleted.

### Not only linker length, but also linker sequence modulates fiber folding

**Figure 8.**
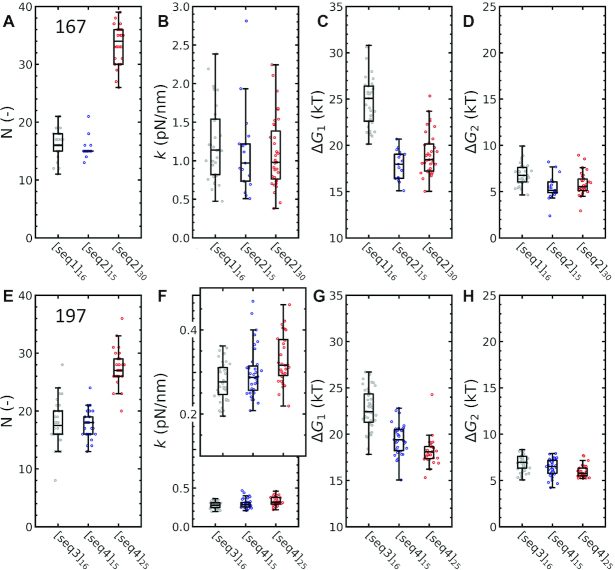
The unstacking energy, but not the stiffness of the fiber, depends on the linker DNA sequence. Force–extension curves of three fibers with either a different linker DNA sequences or a different number of repeats (denoted seq1–4, see Materials and Methods) were fitted to equation 15. (**A**) The number of nucleosomes *N* that were fitted largely reproduced the number of 601 repeats in the 167 NRL DNA substrates. Note that curves from seq2 were more stringently selected for the number of nucleosomes, yielding a narrower distribution. (**B**) The number of 601 repeats did not affect stiffness $k$ (*P* = 0.92) nor did the sequence (*P* = 0.20). (**C**) Linker DNA sequence, however, significantly affected the unstacking energy $\Delta {G_1}$ by approximately 7 k_B_T (*P* < 0.005). The number of nucleosomes did not change $\Delta {G_1}$ (*P* = 0.09). (**D**) Unwrapping energy $\Delta {G_2}$ was not affected by either nucleosome number (*P* = 0.43) or sequence (*P* = 0.05). Experiments on another group of NRL fibers yielded similar trends for (**E**) number of nucleosomes, (**F**) $k$ (seq: *P* = 0.09, *N*: *P* = 0.03), (**G**) $\Delta {G_1}$ (seq: *P* < 0.005, *N*: *P* = 0.05), and (**H**) $\Delta {G_2}$ (seq: *P* = 0.01, *N*: *P* = 0.04).

Since our rbMC simulations suggest that deformation of the linker DNA defines nucleosome stacking, and knowing that the mechanical properties of DNA depend on its sequence, we expect that the sequence of the linker DNA may play an important role in the stability of chromatin fiber folding. Two batches of chromatin fibers with NRL 167, consisting of different linker DNA sequences but a similar number of repeats were reconstituted, stretched, and fitted with the statistical mechanics model. They were compared with another batch of chromatin fibers with identical linker sequence, but with a different number of repeats of the 601 sequence. The fitted parameters are depicted in Figure 8A–D. A similar approach was followed for three batches of chromatin fibers with NRL 197, depicted in Figure 8E–H. We observed that the difference in linker DNA sequence (*see* Materials and Methods) did not affect the measured stiffness of the fiber, indicating the same folding. All fits yielded approximately the same $\Delta {G_2}.$ The rupture energy$\ \Delta {G_1}$, however, differed significantly: ∼6 k_B_T (*P* < 0.001). Despite that the stiffness of the folded fibers did not depend on the linker DNA sequence, the rupture force did. The difference in $\Delta {G_1}$ should predominantly be attributed to the different sequence-dependent mechanical properties of the linker DNA. This modulation of stability of the folded fiber could play a role in epigenetic regulation *in vivo*. However, a full understanding of how the linker DNA sequence directs higher-order folding requires studying linker sequence variations in a more systematic manner, which will be explored in future work.

## DISCUSSION

The role of the linker DNA on the topology of chromatin fibers has been debated extensively, but generally in a coarse and qualitative manner. Here, we combined computational modeling with experimental single-molecule force spectroscopy and studied the effects of single base pair increments on chromatin fiber folding. Using rbMC, we showed that the energy cost of deforming the linker DNA to enable nucleosome stacking decreased with increasing linker length, as the resulting stress could be distributed over a larger number of base pairs. The linker DNA energy in stacked nucleosomes was generally dominated by contributions of the rotational step parameters. We showed that the twist of the linker DNA is an important factor in fiber folding, demonstrated by the 10 base pair periodicity in 1-start fibers, where the most favorable states were approximately 10 k_B_T lower in energy than the most unfavorable ones. A similar 10 bp periodicity was observed in nucleosome stacking in 2-start fibers. We conclude that 1-start fibers preferred to twist the linker DNA, where 2-start fibers compromised stacking when the length of the linker was not optimal.

Experimentally, we measured a 10 bp modulation in stiffness and stacking energy of folded fibers for both short and long linker lengths. In fibers with short linker DNA, the modulations inhibited stacking between nucleosomes, which is apparent from the reduced stiffness and rupture energy. For linker DNA between 45 and 55 bp, the modulations were smoother, indicating that longer linker DNA can more easily accommodate the higher-order structure imposed by nucleosome stacking. The periodic modulation in the experimental data qualitatively matched with the rbMC simulations, though the phasing appears off: the rbMC simulations suggest that fibers with 10*n* + 5 bp of linker DNA yield the most stable stacking interactions, while the experimental data show the strongest stacking interactions for NRLs with 10*n* bp of linker DNA. The somewhat naive implementation of the nucleosome-nucleosome interaction potential may be responsible for this difference. In principle, the simulations could be used to reproduce the entire force–extension curves that we measured. However, previous work showed that the current implementation of the simulations could not reach equilibrium conditions for nucleosome unstacking (33), which is also observed experimentally for these transitions. Moreover, the harmonic nucleosome-nucleosome interaction potential used in our rbMC model is probably underestimating the energy for larger deviations from the equilibrium geometry. The very small periodic modulation in stacking energy for short NRLs is therefore likely an underestimation. A more confined stacking interaction increases the energy for non-optimal stacking configurations and would differentiate stronger between viable and non-viable states. The experimental curves on short NRLs indeed featured a more abrupt inhibition of stacking.

Based on the size of the rupture plateau, we interpret the first transition as unstacking of nucleosome–nucleosome interactions. The extension per nucleosome of the first unstacked nucleosome conformation (conformation II in Figure 5A) suggests that the nucleosomes in this conformation have 92 bp of wrapped DNA. However, fitting a WLC with a persistence length of 50 nm to this conformation may not be accurate as the deflection of the path of the DNA that results from such wrapping may require more detailed account of the geometry (41). Whether the nucleosomes are fully wrapped in the stacked conformation or that the nucleosomes are already partially unwrapped in the stacked state to relieve some of the stress in the linker DNA could therefore not be extracted from the experimental data. The MC simulations suggest that unwrapping does not occur in stacked nucleosomes. But this result depends on the chosen value for the wrapping energy. Experimentally the (lack of) unwrapping in stacked nucleosomes could be checked using FRET experiments, similar to those by Kilic *et al.* (42), with appropriate label positions. Pending such experimental validation, $\Delta {G_1}$ should be interpreted with caution.

Our finding that fiber folding is optimal, and consequently that energy is minimized, for linker lengths of multiples of 10 does not imply that these will more prominent *in vivo*. In fact, a 10*n* + 5 periodicity was shown to be most prominent in yeast (43). Cells may organize (part of) their nucleosomes such that nucleosome stacking is impeded. This gives chromatin remodelling enzymes a means to indirectly control higher order folding.

Previously reported values of the nucleosome-nucleosome interaction energy, as fitted from force spectroscopy data, varied between 2.5 and 18 k_B_T (44,45). The fitted rupture energies here are within that range. However, the fitted rupture energies cannot be compared directly with the energies that were used as inputs for the rbMC simulations. The rupture energies that we fitted represent the difference in free energy between stacked and unstacked nucleosomes, including all contributions, such as deformation of the nucleosomal DNA, histone–DNA interactions, DNA–DNA repulsion and deformation of the linker DNA. Unfortunately, this does not yield insight in the exact structure of the nucleosomes in each conformation and we cannot tell from end-to-end distance if stacked nucleosomes are fully wrapped, or not. In the rbMC simulations on the other hand, the energies were assigned to specific contacts, and deformation energies of the linker DNA were implicitly modeled from the standard deviation of the step parameters and computed afterward. Nevertheless, both the fitted unstacking and partial unwrapping energies were in roughly in range with the input parameters of the rbMC simulations.

Nucleosome–nucleosome stacking is thought to be primarily, but not exclusively, mediated by interactions between the H4 histone tail and its adjacent nucleosome. Future experiments with gH4 histones with different linker lengths may confirm and refine this insight. To improve our rbMC model, we therefore propose that stacking can better be described by multiple freely-jointed chains that represent the histone tails, rather than a harmonic nucleosome–nucleosome interaction potential. The H4-deletion experiments on NRL 197 fibers suggest however that additional histone interactions play a role in nucleosome stacking. The large and positively charged H3 tail is likely to interact with the negatively charged linker DNA for example (42,46). This may severely affect the mechanical properties of the DNA and would invalidate the use of standard step parameters for rbMC simulations of linker DNA. Similar effects can be anticipated for linker histones and other additional DNA organizing proteins, that we did not include in this study. The rather large impact of the linker DNA on the orientation of adjacent nucleosomes, e.g. 36° of twist per base pair, probably makes that a harmonic interaction potential can still reproduce major experimental features.

To date, structural data on nucleosome-nucleosome stacking predominantly show short-range interactions that feature close packing of interacting nucleosomes (47–50) similar to the packing in crystal structures (51). These data were generally obtained using nucleosome core particles rather than chromatin fibers, which lifts geometrical constraints of the linker DNA. Experimental data on fibers have only reported close-packing in tetra-nucleosomes (5,6), showing that such constraints may compromise stacking. It should also be taken into account that the buffer conditions that are required for successfully obtaining high-resolution structural data may drive the fiber into particular states. Here, we measured in buffer conditions that mimic *in vivo* conditions. Interestingly, recent single-molecule FRET experiments on NRL 167 chromatin fibers reported a very dynamic arrangement, that was interpreted as rapid changes in the register of tetranucleosomal fiber sub-structures (52), highlighting the transient nature of nucleosome stacking interactions. This more dynamic and on average less tightly packed structure is fully consistent with our current findings.

The ability to account for minimal differences in composition between fibers, as well as the ability to resolve different mechanical aspects of fiber folding, like stiffness, rupture energy and cooperativity of unfolding, gives single-molecule force spectroscopy a competitive edge with respect to bulk techniques like sedimentation velocity measurements (32,53,54), native gel electrophoresis (55) and small angle X-ray scattering (6,56). Furthermore, although only a single dimension of the fiber is measured, the nanometer accuracy and the direct manipulation of individual fibers yields quantitative, structural insight. We show here that sufficiently refined coarse-grain modeling provides a powerful combination that helps to resolve the dynamic structure of chromatin higher-order folding.

It is remarkable that our statistical mechanics model, with only three free fitting parameters, could capture all the different fiber topologies and compositions. Previously, we have only validated the model for two NRLs (10,11,31). Despite our best efforts to maintain the integrity of the fibers, we observed some variation in fiber fit parameters between fibers reconstituted from DNA with the same sequence. A large fraction of these variations was captured by taking into account the number of nucleosomes. Nevertheless, there was some correlation between $k$ and $\Delta {G_1}$ within each NRL, as depicted in Supplementary Figure S2. Interestingly, the correlation for NRL 167, NRL 176 and NRL 177 was relatively small: $R\ = \ 0.46.$ 197 NRL fibers had a larger correlation coefficient: $R\ = \ 0.82$. Ideally, $k$ and $\Delta {G_1}$ would be uncorrelated since the statistical mechanics model should separate mechanical properties from fiber composition. However, when fibers are not fully saturated, defects within a fiber would affect fiber folding. Defects at different locations could induce systematic variations that cannot be captured in our statistical physics model, and lift the independence of the fit parameters. It is noteworthy that the fibers that were attributed to fold in a 2-start topology, which corresponds to cooperative (un-)stacking (31), featured the lowest correlation in fit parameters. Such cooperative stacking would reduce both compositional defects, as nucleosomes are better stabilized, and provide additional robustness against stretching and unstacking, having two stacks of nucleosomes in series. Fibers that fold in 1-start topologies would therefore be more susceptible for stacking defects, leading to convoluted fitting parameters. Thus, the larger correlation between the stiffness and the first rupture energy may indirectly point to 1-start folding.

By comparing the force–extension curves of equal NRL fibers with different DNA sequence, we showed that mechanical properties of the linker DNA have a defining role in chromatin fiber folding. This may have important consequences *in vivo*. A-tracts, for instance, are predominantly found in the linker DNA regions of chromatin (57). Their higher stiffness and intrinsic curvature drive nucleosomes into flanking positions (58). As a secondary effect, these A-tracts may drive nucleosomes into orientations that can prohibit or enhance stacking, depending on the precise position and length of the A-tract and linker length, and therefore also control higher-order folding. More global effects can also be envisioned: the most abundant linker lengths *in vivo* are 10*n* + 5 (19,21), which we show here to fold into 0-start, open chromatin fibers for short linker lengths. Increasing the linker length promotes 1-start topologies. Nucleosome positioning may thus modulate the accessibility of DNA locally and/or globally. It can result in direct effects, by wrapping the DNA in a nucleosome, or indirect effects, by controlling nucleosome stacking and the higher-order structure of the chromatin fiber.

The debate on the role, abundance and existence of the 30 nm chromatin fiber *in vivo*, has not settled. Current experimental data, based on sophisticated EM and chromatin capture techniques, tend towards dismissing such structures (2). In this study we show, that especially for short NRLs, a nucleosome shift of a single base pair can make the difference between stacked and non-stacked nucleosomes. The recent study by Baldi *et al.* (19) suggests that regular NRLs are more prominent than previously thought. However, one would need single base pair positioning accuracy for multiple nucleosomes in individual chromatin fragments to determine if this affects fiber formation and current technology does not allow for such accuracy. Pending such data, it is more likely to assume that irregular linker lengths result in irregular nucleosome stacking patterns along the fiber. Single-molecule force spectroscopy experiments on natively assembled chromatin (59) showed that despite such anticipated irregularity nucleosome stacking is prominent. It is therefore more compelling to discuss chromatin folding in terms of nucleosome stacking pairs that are defined by, amongst other features, the linker length, rather than imposing long-range regularity to understand chromatin higher-order structure (60).

The sequence-dependence of nucleosome stacking, both by the positioning of nucleosome pairs and by modulation of the bendability and twistability of the linker DNA, brings up an intriguing possibility. Next to post-translational histone modifications, and remodeler-induced nucleosomal rearrangements, the DNA sequence itself may partially encode its (lack of) higher-order structure. Therefore, our genome may have evolved to mechanically favor particular higher-order organizations. Future studies will need to verify this hypothesis. Here we show that such features require single base pair accuracy, as a shift of a nucleosome with one base pair can make a major difference in chromatin higher-order structure.

## Supplementary Material

gkab058_Supplemental_File

## References

[1] Poirier M.G. , Bussiek M., Langowski J., Widom J. Spontaneous access to DNA target sites in folded chromatin fibers. J. Mol. Biol. 2008; 379:772–786. 18485363 10.1016/j.jmb.2008.04.025 PMC2481406

[2] Fierz B. , Poirier M.G. Biophysics of chromatin dynamics. Annu. Rev. Biophys. 2019; 48:345–321. 10.1146/annurev-biophys-070317-032847 30883217

[3] Wolffe A.P. Gene regulation: insulating chromatin. Curr. Biol. 1994; 4:85–87. 7922323 10.1016/s0960-9822(00)00022-1

[4] Luger K. , Dechassa M.L., Tremethick D.J. New insights into nucleosome and chromatin structure: an ordered state or a disordered affair. Nat. Rev. Mol. Cell Biol. 2012; 13:436–447. 22722606 10.1038/nrm3382 PMC3408961

[5] Song F. , Chen P., Sun D., Wang M., Dong L., Liang D., Xu R.-M., Zhu P., Li G. Cryo-EM study of the chromatin fiber reveals a double helix twisted by tetranucleosomal units. Science. 2014; 344:376–380. 24763583 10.1126/science.1251413

[6] Schalch T. , Duda S., Sargent D.F., Richmond T.J. X-ray structure of a tetranucleosome and its implications for the chromatin fibre. Nature. 2005; 436:138–141. 16001076 10.1038/nature03686

[7] Risca V.I. , Denny S.K., Straight A.F., Greenleaf W.J. Variable chromatin structure revealed by in situ spatially correlated DNA cleavage mapping. Nature. 2017; 541:237–241. 28024297 10.1038/nature20781 PMC5526328

[8] Hsieh T.-H.S. , Weiner A., Lajoie B., Dekker J., Friedman N., Rando O.J. Mapping nucleosome resolution chromosome folding in yeast by Micro-C. Cell. 2015; 162:108–119. 26119342 10.1016/j.cell.2015.05.048 PMC4509605

[9] Robinson P.J.J. , Fairall L., Huynh V.A.T., Rhodes D EM measurements define the dimensions of the ‘“30-nm”’ chromatin fiber: evidence for a compact, interdigitated structure. Proc. Natl. Acad. Sci. U.S.A. 2006; 103:6506–6511. 16617109 10.1073/pnas.0601212103 PMC1436021

[10] Kruithof M. , Chien F.-T., Routh A., Logie C., Rhodes D., van Noort J. Single-molecule force spectroscopy reveals a highly compliant helical folding for the 30-nm chromatin fiber. Nat. Struct. Mol. Biol. 2009; 16:534–540. 19377481 10.1038/nsmb.1590

[11] Kaczmarczyk A. , Allahverdi A., Brouwer T.B., Nordenskiöld L., Dekker N.H., van Noort J. Single-molecule force spectroscopy on histone H4 tail-cross-linked chromatin reveals fiber folding. J. Biol. Chem. 2017; 292:17506–17513. 28855255 10.1074/jbc.M117.791830 PMC5655525

[12] Maeshima K. , Ide S., Babokhov M. Dynamic chromatin organization without the 30-nm fiber. Curr. Opin. Cell Biol. 2019; 58:95–104. 30908980 10.1016/j.ceb.2019.02.003

[13] Ou H.D. , Phan S., Deerinck T.J., Thor A., Ellisman M.H., O’Shea C.C. ChromEMT: Visualizing 3D chromatin structure and compaction in interphase and mitotic cells. Science. 2017; 357:eaag0025. 28751582 10.1126/science.aag0025 PMC5646685

[14] Maeshima K. , Ide S., Hibino K., Sasai M. Liquid-like behavior of chromatin. Curr. Opin. Genet. Dev. 2016; 37:36–45. 26826680 10.1016/j.gde.2015.11.006

[15] Ricci M.A. , Manzo C., García-Parajo M.F., Lakadamyali M., Cosma M.P. Chromatin fibers are formed by heterogeneous groups of nucleosomes in vivo. Cell. 2015; 160:1145–1158. 25768910 10.1016/j.cell.2015.01.054

[16] Brogaard K. , Xi L., Wang J.-P., Widom J. A map of nucleosome positions in yeast at base-pair resolution. Nature. 2012; 486:496–501. 22722846 10.1038/nature11142 PMC3786739

[17] Segal E. , Fondufe-Mittendorf Y., Chen L., Thåström A., Field Y., Moore I.K., Wang J.-P.Z., Widom J. A genomic code for nucleosome positioning. Nature. 2006; 442:772–778. 16862119 10.1038/nature04979 PMC2623244

[18] Jiang C. , Pugh B.F. Nucleosome positioning and gene regulation: advances through genomics. Nat. Rev. Genet. 2009; 10:161–172. 19204718 10.1038/nrg2522 PMC4860946

[19] Baldi S. , Krebs S., Blum H., Becker P.B. Genome-wide measurement of local nucleosome array regularity and spacing by nanopore sequencing. Nat. Struct. Mol. Biol. 2018; 25:894–901. 30127356 10.1038/s41594-018-0110-0

[20] Clark S.C. , Chereji R. V., Lee P.R., Fields R.D., Clark D.J. Differential nucleosome spacing in neurons and glia. Neurosci. Lett. 2019; 714:134559. 31639421 10.1016/j.neulet.2019.134559 PMC6943982

[21] Widom J. A relationship between the helical twist of DNA and the ordered positioning of nucleosomes in all eukaryotic cells. Proc. Natl. Acad. Sci. U.S.A. 1992; 89:1095–1099. 1736292 10.1073/pnas.89.3.1095 PMC48392

[22] Perišić O. , Collepardo-Guevara R., Schlick T. Modeling studies of chromatin fiber structure as a function of DNA linker length. J. Mol. Biol. 2010; 403:777–802. 20709077 10.1016/j.jmb.2010.07.057 PMC2966533

[23] Koslover E.F. , Fuller C.J., Straight A.F., Spakowitz A.J. Local geometry and elasticity in compact chromatin structure. Biophys. J. 2010; 99:3941–3950. 21156136 10.1016/j.bpj.2010.10.024 PMC3000514

[24] Eslami-Mossallam B. , Schram R.D., Tompitak M., van Noort J., Schiessel H. Multiplexing genetic and nucleosome positioning codes: a computational approach. PLoS One. 2016; 11:e0156905. 27272176 10.1371/journal.pone.0156905 PMC4896621

[25] Bass M.V , Nikitina T., Norouzi D., Zhurkin V.B., Grigoryev S.A. Nucleosome spacing periodically modulates nucleosome chain folding and DNA topology in circular nucleosome arrays. J. Biol. Chem. 2019; 294:4233–4246. 30630950 10.1074/jbc.RA118.006412 PMC6422092

[26] Norouzi D. , Zhurkin V.B. Dynamics of chromatin fibers: comparison of monte carlo simulations with force spectroscopy. Biophys. J. 2018; 115:1644–1655. 30236784 10.1016/j.bpj.2018.06.032 PMC6225046

[27] Norouzi D. , Zhurkin V.B. Topological polymorphism of the two-start chromatin fiber. Biophys. J. 2015; 108:2591–2600. 25992737 10.1016/j.bpj.2015.04.015 PMC4457244

[28] Lowary P.T. , Widom J. New DNA sequence rules for high affinity binding to histone octamer and sequence-directed nucleosome positioning. J. Mol. Biol. 1998; 276:19–42. 9514715 10.1006/jmbi.1997.1494

[29] Wu C. , Read C., McGeehan J., Crane-Robinson C. The construction of customized nucleosomal arrays. Anal. Biochem. 2016; 496:71–75. 26706802 10.1016/j.ab.2015.11.018

[30] Ekundayo B. , Richmond T.J., Schalch T. Capturing structural heterogeneity in chromatin fibers. J. Mol. Biol. 2017; 429:3031–3042. 28893533 10.1016/j.jmb.2017.09.002

[31] Meng H. , Andresen K., van Noort J. Quantitative analysis of single-molecule force spectroscopy on folded chromatin fibers. Nucleic Acids Res. 2015; 43:3578–3590. 25779043 10.1093/nar/gkv215 PMC4402534

[32] Correll S.J. , Schubert M.H., Grigoryev S.A. Short nucleosome repeats impose rotational modulations on chromatin fibre folding. EMBO J. 2012; 31:2416–2426. 22473209 10.1038/emboj.2012.80 PMC3364735

[33] de Jong B.E. , Brouwer T.B., Kaczmarczyk A., Visscher B., van Noort J. Rigid basepair monte carlo simulations of one-start and two-start chromatin fiber unfolding by force. Biophys. J. 2018; 115:1848–1859. 30366627 10.1016/j.bpj.2018.10.007 PMC6303278

[34] Buckwalter J.M. , Norouzi D., Harutyunyan A., Zhurkin V.B., Grigoryev S.A. Regulation of chromatin folding by conformational variations of nucleosome linker DNA. Nucleic Acids Res. 2017; 45:9372–9387. 28934465 10.1093/nar/gkx562 PMC5766201

[35] Eslami-Mossallam B. , Schiessel H., van Noort J. Nucleosome dynamics: sequence matters. Adv. Colloid Interface Sci. 2016; 232:101–113. 26896338 10.1016/j.cis.2016.01.007

[36] Brouwer T.B. , Kaczmarczyk A., Pham C., van Noort J. Unraveling DNA Organization with Single-Molecule Force Spectroscopy Using Magnetic Tweezers. 2018; NY Humana Press 317–349. 10.1007/978-1-4939-8675-0_17 30109618

[37] Flaus A. Principles and practice of nucleosome positioning *in vitro*. *Front*. Life Sci. 2011; 5:5–27.

[38] Kaczmarczyk A. , Brouwer T.B., Pham C., Dekker N.H., van Noort J Probing chromatin structure with magnetic tweezers. Methods Mol. Biol. 2018; 1814:297–323. 29956240 10.1007/978-1-4939-8591-3_18

[39] Klinker H. , Haas C., Harrer N., Becker P.B., Mueller-Planitz F. Rapid purification of recombinant histones. PLoS One. 2014; 9:e104029. 25090252 10.1371/journal.pone.0104029 PMC4121265

[40] Koopmans W.J.A. , Buning R., Schmidt T., van Noort J. spFRET using alternating excitation and FCS reveals progressive DNA unwrapping in nucleosomes. Biophys. J. 2009; 97:195–204. 19580757 10.1016/j.bpj.2009.04.030 PMC2711358

[41] Kulić I.M. , Mohrbach H., Lobaskin V., Thaokar R., Schiessel H. Apparent persistence length renormalization of bent DNA. Phys. Rev. E - Stat. Nonlinear, Soft Matter Phys. 2005; 72:041905. 10.1103/PhysRevE.72.041905 16383418

[42] Kilic S. , Felekyan S., Doroshenko O., Boichenko I., Dimura M., Vardanyan H., Bryan L.C., Arya G., Seidel C.A.M., Fierz B Single-molecule FRET reveals multiscale chromatin dynamics modulated by HP1α. Nat. Commun. 2018; 9:235. 29339721 10.1038/s41467-017-02619-5 PMC5770380

[43] Wang J.-P. , Fondufe-Mittendorf Y., Xi L., Tsai G.-F., Segal E., Widom J. Preferentially quantized linker DNA lengths in *Saccharomyces cerevisiae*. PLoS Comput. Biol. 2008; 4:e1000175. 18787693 10.1371/journal.pcbi.1000175 PMC2522279

[44] Cui Y. , Bustamante C. Pulling a single chromatin fiber reveals the forces that maintain its higher-order structure. Proc. Natl. Acad. Sci. U.S.A. 2000; 97:127–132. 10618382 10.1073/pnas.97.1.127 PMC26627

[45] Meng H. , Andresen K., Van Noort J. Quantitative analysis of single-molecule force spectroscopy on folded chromatin fibers. Nucleic Acids Res. 2015; 43:3578–3590. 25779043 10.1093/nar/gkv215 PMC4402534

[46] Li Z. , Kono H. Distinct roles of histone H3 and H2A tails in nucleosome stability. Sci. Rep. 2016; 6:31437. 27527579 10.1038/srep31437 PMC4985630

[47] Funke J.J. , Ketterer P., Lieleg C., Korber P., Dietz H. Exploring nucleosome unwrapping using DNA origami. Nano Lett. 2016; 16:7891–7898. 27960448 10.1021/acs.nanolett.6b04169

[48] Funke J.J. , Ketterer P., Lieleg C., Schunter S., Korber P., Dietz H. Uncovering the forces between nucleosomes using DNA origami. Sci. Adv. 2016; 2:e1600974. 28138524 10.1126/sciadv.1600974 PMC5262459

[49] Eltsov M. , Grewe D., Lemercier N., Frangakis A., Livolant F., Leforestier A. Nucleosome conformational variability in solution and in interphase nuclei evidenced by cryo-electron microscopy of vitreous sections. Nucleic Acids Res. 2018; 46:9189–9200. 30053160 10.1093/nar/gky670 PMC6158616

[50] Mangenot S. , Raspaud E., Tribet C., Belloni L., Livolant F. Interactions between isolated nucleosome core particles: a tail-bridging effect. Eur. Phys. J. E. 2002; 7:221–231.

[51] Korolev N. , Lyubartsev A.P., Nordenskiöld L. A systematic analysis of nucleosome core particle and nucleosome-nucleosome stacking structure. Sci. Rep. 2018; 8:1543. 29367745 10.1038/s41598-018-19875-0 PMC5784010

[52] Fierz B. Dynamic chromatin regulation from a single molecule perspective. ACS Chem. Biol. 2016; 11:609–620. 26565113 10.1021/acschembio.5b00832

[53] Grigoryev S.A. , Arya G., Correll S., Woodcock C.L., Schlick T. Evidence for heteromorphic chromatin fibers from analysis of nucleosome interactions. Proc. Natl. Acad. Sci. U.S.A. 2009; 106:13317–13322. 19651606 10.1073/pnas.0903280106 PMC2726360

[54] Woodcock C.L. , Grigoryev S.A., Horowitz R.A., Whitaker N. A chromatin folding model that incorporates linker variability generates fibers resembling the native structures. Proc. Natl. Acad. Sci. U.S.A. 1993; 90:9021–9025. 8415647 10.1073/pnas.90.19.9021 PMC47493

[55] Dorigo B. , Schalch T., Kulangara A., Duda S., Schroeder R.R., Richmond T.J., Xu R.-M., Zhu P., Li G. Nucleosome arrays reveal the two-start organization of the chromatin fiber. Science. 2004; 306:1571–1573. 15567867 10.1126/science.1103124

[56] Maeshima K. , Imai R., Hikima T., Joti Y. Chromatin structure revealed by X-ray scattering analysis and computational modeling. Methods. 2014; 70:154–161. 25168089 10.1016/j.ymeth.2014.08.008

[57] Cui F. , Zhurkin V.B. Distinctive sequence patterns in metazoan and yeast nucleosomes: implications for linker histone binding to AT-rich and methylated DNA. Nucleic Acids Res. 2009; 37:2818–2829. 19282449 10.1093/nar/gkp113 PMC2685081

[58] Segal E. , Widom J. Poly(dA:dT) tracts: major determinants of nucleosome organization. Curr. Opin. Struct. Biol. 2009; 19:65–71. 19208466 10.1016/j.sbi.2009.01.004 PMC2673466

[59] Hermans N. , Huisman J.J., Brouwer T.B., Schächner C., van Heusden G.P.H., Griesenbeck J., van Noort J. Toehold-enhanced LNA probes for selective pull down and single-molecule analysis of native chromatin. Sci. Rep. 2017; 7:16721. 29196662 10.1038/s41598-017-16864-7 PMC5711847

[60] Cai S. , Böck D., Pilhofer M., Gan L. The in situ structures of mono-, di-, and trinucleosomes in human heterochromatin. Mol. Biol. Cell. 2018; 29:2450–2457. 30091658 10.1091/mbc.E18-05-0331 PMC6233054

