# Supplementary material for: A critical role for linker DNA in higher-order folding of chromatin fibers
Source: Nucleic Acids Res. 2021 Feb 15;49(5):2537–51. doi: 10.1093/nar/gkab058 (PMC7969035; doi:10.1093/nar/gkab058)
Supplement: gkab058_Supplemental_File [file gkab058_supplemental_file.pdf]

Supplementary material

rbMC simulations

Chromatin fibers were simulated using a rigid base pair model, expanded to include (un)wrapping of nucleosomal DNA and (un)stacking of nucleosomes in one-start and two-start chromatin fibers [1]. For each structure, 1.000 Monte Carlo iterations were calculated while the fibers were exposed to 3 pN of force. To speed up the simulations, the contour length was limited to 1800 base pairs, resulting in DNA handles that were shorter than in the experiments. Chromatin fibers in the absence of force, as shown in figure 2-4, were simulated with 8 601 repeats. 10.000 Monte Carlo iterations were calculated, where each step corresponded to sequential replacement and evaluation of every base pair in the DNA tether. Energy penalties were included for steric clashes between nucleosomes and excluded volume effects. Every 200 iterations, an independent structure was stored for further analysis. To represent the typical conformation of a set of nucleosomes, step parameters of all 10.000 independent states were averaged. The standard deviations were calculated from the energies of all stored states. The total energy was calculated as the sum of the base pair step energies in the 6 degrees of freedom plus the wrapping and stacking energies. The energies depicted in figure 4 were calculated relative to the 0-start fiber.

DNA sequences

NRL 167x16 - NRL 177x16 and NRL 192x16 - NRL 202x16 series All plasmids in this series share the 601 nucleosome positioning sequence [2] (underlined) and a basis linker sequence of 20 base pairs, optionally extended in single base pair steps with the sequence in lowercase:

[CTGGAGAATCCCGGTGCCGAGGCCGCTCAATTGGTCGTAGCAAGCTCTAGCACCGCTTAAACGCACGTAC
GCGCTGTCCCCCGCGTTTTAACCGCCAAGGGGATTACTCCCTAGTCTCCAGGCACGTGTCAGATATATACA
TCCTGTctgactggaccctatacgcggtattgaatcgacacCTTGCCACCCCGGGCTGTGA]$_{16}$

Linker sequence comparison For comparing the effect of the linker DNA sequence on fiber folding the following substrates were used.

16 repeats of NRL 167 with linker DNA sequence 1:

[CTGGAGAATCCCGGTGCCGAGGCCGCTCAATTGGTCGTAGCAAGCTCTAGCACCGCTTAAACGCACGTAC
GCGCTGTCCCCCGCGTTTTAACCGCCAAGGGGATTACTCCCTAGTCTCCAGGCACGTGTCAGATATATACA
TCCTGTCTTGCCACCCCGGGCTGTGA]$_{16}$

15 and 30 repeats of NRL 167 with linker DNA sequence 2:

[CTGGAGAATCCCGGTGCCGAGGCCGCTCAATTGGTCGTAGCAAGCTCTAGCACCGCTTAAACGCACGTAC
GCGCTGTCCCCCGCGTTTTAACCGCCAAGGGGATTACTCCCTAGTCTCCAGGCACGTGTCAGATATATACA
TCCTGTGCATGTACTCGGGGGCCGCC]$_{15/30}$

16 repeats of NRL 197 with linker DNA sequence 3:

[CTGGAGAATCCCGGTGCCGAGGCCGCTCAATTGGTCGTAGCAAGCTCTAGCACCGCTTAAACGCACGTAC
GCGCTGTCCCCCGCGTTTTAACCGCCAAGGGGATTACTCCCTAGTCTCCAGGCACGTGTCAGATATATACA
TCCTGTTGGACCCTATACGCGGTATTGAATCGACACCTTGCCACCCCGGGCTGTGA]$_{16}$

15 and 25 repeats of NRL 197 with linker DNA sequence 4:

[CTGGAGAATCCCGGTGCCGAGGCCGCTCAATTGGTCGTAGCAAGCTCTAGCACCGCTTAAACGCACGTACGCGCTGTCCCCCGCGTTTTAACCGCCAAGGGGATTACTCCCTAGTCTCCAGGCACGTGTCAGATATATACATCCTGTGCATGTATTGAACAGCGACCTTCTCGGGATGGACCCTATACGCGGCCGCC]$_{15/25}$

Magnetic tweezers setup

A multiplexed magnetic tweezers setup equipped with a NIKON CFI Plan Fluor objective MRH01401 (NA = 1.3, 40x, Oil, *NIKON Corporation*, Tokyo, Japan) was used to stretch our chromatin fibers. The field-of-view (FOV) was captured on a 25 Mpix Condor camera (cmv5012-F30-S[D]-M[F]-P8, *CMOS Vision GmbH*, Schaffhausen, Switzerland) using an infinity-corrected tube lens ITL200 (*Thorlabs*, Newton, USA). The flow cell was illuminated with a 100 mW, 645 nm collimated LED (LED-1115-ELC-645-29-2, *IMM Photonics GmbH*, Unterschleißheim, Germany). The large FOV allowed for hundreds of fibers to be measured simultaneously. The camera was connected to a PCIe-1433 (*National Instruments*, Austin, USA) plugged in a T7610 PC (*Dell*, Round Rock, USA) equipped with a ten-core Intel Xeon 2.8 GHz processor (E5-2680 v2, *Intel*, Santa Clara, USA) and 32GB DDR3 memory. The setup measured the full FOV at 30 fps. Samples were measured in custom-built flow cells, mounted on a multi-axis piezo scanner P-517.3CL (*Physik Instrumente GmbH & Co. KG*, Karlsruhe, Germany). Forces were exerted by a pair of N50 5 mm cube magnets (W-05-N50-G, *Supermagnete*, Webcraft GmbH, Gottmadingen, Germany). Force F was calculated using:

$$F = A\left(0.7\exp\left(-\frac{h}{c_1}\right) + 0.3\exp\left(-\frac{h}{c_2}\right)\right), \quad (1)$$

where h was the magnet height, and $c_1 = 1.4$ mm and $c_2 = 0.8$ mm were decay lengths describing the double-exponential decay of the magnetic field and $A = 85$ pN for 2.8 µm paramagnetic beads (Dynabeads M270 Streptavidin, *Thermo Fisher Scientific*). Forces were calibrated using equipartition theorem, as previously described [3–5].

Statistical mechanics model

To infer mechanical properties of individual chromatin fibers the force-extension curves were fitted to a statistical mechanics model developed by Meng *et al.* [6]. The model reduced chromatin fibers to their fundamental units: individual nucleosomes that could either be in a stacked conformation (I), partially unwrapped conformation (II), singly wrapped conformation (III), and fully unwrapped conformation (IV), as shown in figure 4a. Each conformation i had a corresponding extension and free energy which defines the thermodynamic equilibrium.

The probability $P_i(f)$ that a nucleosome is in conformation i was defined as:

$$P_i(f) = \frac{1}{Z}\exp\left(-\frac{g_\mathrm{i}(f)+W}{k_\mathrm{B}T}\right), \quad (2)$$

where the partition function was defined as $Z = \sum_i \exp(-g_\mathrm{i}(f)/k_\mathrm{B}T)$ [7], and $g_\mathrm{i}(f, z_i)$ represents the free energy of conformation i. W is the work done by the bead corresponding to:

$$W = \int_0^z f(\tilde{z})\mathrm{d}\tilde{z} = fz - \int_0^f f(\tilde{f})\mathrm{d}\tilde{f} \quad (3)$$

The nucleosomes are strongly compacted in the stacked conformation (conformation I). The extension of this conformation z_fiber under force f was described by a Hookean spring:

$$z_\mathrm{fiber}(f) = \frac{f}{k} + z_0, \quad (4)$$

where k described the stiffness of the chromatin fiber and z_0 the extension of the folded fiber at $f = 0$ pN. z_0 was fixed at a nucleosome line density of 1.5 nm. The free energy of the fiber conformation was defined as:

$$g_{\text{fiber}}(f) = f z_{\text{fiber}}(f) - \int_0^f z_{\text{fiber}}(\tilde{f})\mathrm{d}\tilde{f} = f z_{\text{fiber}}(f) - \frac{f^2}{2k}. \qquad (5)$$

It required rupture energy ΔG_1 to break the stacking interactions, unfold the fiber, and unwrap the outer turn of DNA from the histone core, yielding the partially unwrapped conformation (conformation II).

In the partially unwrapped conformation, the extension per nucleosome was dominated by the stretching of the bare DNA that was wrapped around the histone core, and could therefore be described by a worm-like chain WLC:

$$z_{\text{WLC}}(f, L) = L\left(1 - \frac{1}{2}\sqrt{\frac{k_{\text{B}}T}{fP}} + \frac{f}{S}\right), \qquad (6)$$

where L is the contour length, P the persistence length, and S the stretch modulus of the DNA ($P = 50$ nm, $S = 1000$ pN [8–10]). In this partially unwrapped conformation, we set L_1 = NRL - (147 - 55) bp per nucleosome.

The free energy of a stretched DNA molecule yields:

$$g_{\text{WLC}}(f, L) = f z_{\text{DNA}}(f) - \int z_{\text{WLC}}(f)\mathrm{d}f = f z_{\text{DNA}}(f) - L\left(f - \sqrt{\frac{f \cdot k_B T}{P}} + \frac{f^2}{2S}\right). \qquad (7)$$

Therefore, the free energy of a nucleosome in the partially unwrapped conformation follows:

$$g_1(f, L_1) = g_{\text{WLC}}(f, L_1) + \Delta G_1, \qquad (8)$$

The extension of a singly wrapped nucleosome was calculated with equation 6 using $L_2 =$ NRL – (147 – 55 – 15) bp and free energy:

$$g_2(f, L_2) = g_{\text{WLC}}(f, L_2) + \Delta G_1 + \Delta G_2, \qquad (9)$$

where ΔG_2 was the interaction energy of the contact points broken in this transition.

The extension of the fully unwrapped conformation is equal to the extension of a DNA fragment with a length equal to the NRL, hence, $L_3 =$ NRL [1, 11]. The free energy of this conformation was calculated by:

$$g_3(f, L_3) = g_{\text{WLC}}(f, L_3) + \Delta G_1 + \Delta G_2 + \Delta G_3, \qquad (10)$$

with ΔG_3 representing the wrapping energy of the inner wrap of DNA. The transition between the singly wrapped conformation and the fully unwrapped conformation was not in thermodynamic equilibrium as apparent from the as a stepwise unfolding transition. Therefore, the change in free energy of this transition could not be captured by the statistical mechanics model.

The total extension $Z_{\text{tot}}(f)$ and total free energy $G_{\text{tot}}(f)$ were calculated by a summation of all the extensions and free energies in all conformations i:

$$Z_{\text{tot}}(f) = \sum_i n_i \, z_i(f) \quad (11)$$

$$G_{\text{tot}}(f) = \sum_i n_i \, g_i(f) \quad (12)$$

The force spectroscopy data were fitted with the Boltzmann distribution of conformations to obtain the mechanical properties, the total extension of the tether $< z_{\text{tot}}(f) >$, and the energy changes between the conformations:

$$< z_{\text{tot}}(f) > \;= \frac{1}{Z} \sum_i z_i(f) \exp\left(-\frac{g_i(f)+W}{k_{\text{B}}T}\right). \quad (13)$$

For a chromatin fiber with more than two nucleosomes, the nucleosomes can be distributed over a large, but finite number of states $s = \{n_I, n_{II}, n_{III}, n_{IV}\}$. Multiple indistinguishable states with an equal number of nucleosomes in each of these conformations were accounted for by a degeneracy factor $D(s)$:

$$D(s) = \prod_{i<j} \binom{n_i + n_j}{n_i} \quad (14)$$

where i and j were pairs of conformations in each state. The degeneracy factor was included in the Boltzmann distributions of states:

$$< z_{\text{tot}}(f) > \;= \frac{\sum_s Z_{\text{tot}}(f) \cdot D(s) \cdot \exp(-(G_{tot}(f)+W)/k_{\text{B}}T)}{\sum_s D(s) \cdot \exp(-(G_{tot}(f)+W)/k_{\text{B}}T)} \quad (15)$$

Note that the unwrapping lengths were adjusted since the introduction of the model in 2015. Careful quantification of the step size by Kaczmarczyk *et al.* [12] revealed that 77 base pairs unfold in the transition from conformation III to conformation IV, indicating the unfolding of a complete turn of DNA. Accordingly, to maintain the same extension in the transition between conformations partially unwrapped and singly wrapped conformation, the partially unwrapped conformation was attributed to constrains 135 base pairs [13]. This resolves the nature of the previously unknown 'extended conformation'. Two histone-DNA contact points must be broken in this second unfolding transition, which should account for the corresponding of ΔG_2. In summary, the statistical mechanics model did not change, but the attribution of the number of wrapped base pairs in the two intermediate nucleosome conformations and the interpretation of the difference in free energy between them was different, and the names of these conformations were accordingly changed to partially unwrapped and single wrapped nucleosomes.

Fitting procedure

Prior to fitting the statistical mechanics model, thermal drift was accounted for by subtraction of a linear drift that was fitted from the low force parts ($f < 0.1$ pN) at the beginning and the end of each measurement. Drift typically did not exceed 1 nm/s. By aligning the force-extension curve at $f > 40$ pN with a WLC corresponding the contour length of the DNA substrate, the offset in z was determined. Next, the number of 25 nm steps at $f > 10$ pN, corresponding to the last unwrapping transition, was counted to find the total number of nucleosomal particles reconstituted on the DNA template. The number of tetrasomes was subsequently fit to the statistical physics model in the force range $0.5 < f < 2.5$ pN. In this force range, nucleosomes remain stably folded and/or stacked, so excess extension should be attributed to tetrasomes [6].

Finally, fiber unfolding was fit in the range 0.5 pN $< f < 10$ pN with three free parameters: stiffness k, rupture energy ΔG_1 and partial unwrapping energy ΔG_2. The degeneracy $D(s)$ was fixed to either

equation 14 (degenerate transitions indicating a 1-start fiber) or 1 (cooperative transitions indicating a 2-start fiber), depending on the best fit and was imposed on all fibers of a particular NRL.

The fitted parameters were plotted in a decorated boxplot. The center bar represents the median of the data (Q2), the edges of the box the medians of the split populations (Q1 and Q3); referred to as the interquartile range (IQR). The whiskers of the box plot described the spread of the data: Q1 – 1.5 * IQR to Q3 + 1.5 * IQR. Boxplots were decorated with a scatter plot of all data points, offset with random scatter in the x-direction. The p-values were calculated with one-way ANOVA.

NRL (base pairs)	*N*	*k* (pN/nm)	ΔG_1 (k_BT)	ΔG_2 (k_BT)
**167**	15	1.1 ± 0.1	23 ± 1	9 ± 1
**168**	14	0.4 ± 0.1	17 ± 1	6 ± 1
**169**	15	0.4 ± 0.1	17 ± 1	6 ± 1
**172**	15	0.4 ± 0.1	17 ± 1	7 ± 1
**175**	15	0.4 ± 0.1	20 ± 1	7 ± 1
**176**	14	0.8 ± 0.1	26 ± 1	7 ± 1
**177**	16	0.8 ± 0.1	27± 1	8 ± 1
**192**	15	0.2 ± 0.1	17± 1	8 ± 1
**195**	14	0.3 ± 0.1	22± 1	8 ± 1
**197**	15	0.3 ± 0.1	24± 1	8 ± 1
**198**	14	0.3 ± 0.1	26± 1	6 ± 1
**200**	15	0.2 ± 0.1	20± 1	7 ± 1
**202**	14	0.2 ± 0.1	17± 1	6 ± 1

Supplementary table 1

Fit parameters for chromatin fibers in figure 5c-f. The ±-sign indicates standard error of fit.

NRL (base pairs)	k (pN/nm)	ΔG_1 (k_BT)	ΔG_2 (k_BT)
**167**	1.3 ± 0.5	24 ± 3	7 ± 1
**168**	0.5 ± 0.1	16 ± 2	7 ± 1
**169**	0.4 ± 0.1	17 ± 2	7 ± 1
**170**	0.5 ± 0.2	19 ± 2	7 ± 1
**171**	0.5 ± 0.1	19 ± 3	8 ± 1
**172**	0.6 ± 0.2	19 ± 2	7 ± 1
**173**	0.4 ± 0.1	18 ± 2	8 ± 1
**174**	0.6 ± 0.2	20 ± 2	7 ± 1
**175**	0.5 ± 0.2	20 ± 2	7 ± 1
**176**	0.9 ± 0.5	24 ± 3	7 ± 1
**177**	1.0 ± 0.5	25 ± 3	7 ± 1
**192**	0.2 ± 0.1	17 ± 2	7 ± 1
**193**	0.2 ± 0.1	20 ± 2	7 ± 1
**194**	0.3 ± 0.1	21 ± 3	7 ± 1
**195**	0.3 ± 0.1	21 ± 2	7 ± 1
**196**	0.3 ± 0.1	22 ± 2	7 ± 1
**197**	0.3 ± 0.1	23 ± 2	7 ± 1
**198**	0.3 ± 0.1	24 ± 3	7 ± 1
**199**	0.3 ± 0.1	23 ± 3	7 ± 1
**200**	0.3 ± 0.1	21 ± 2	7 ± 1
**201**	0.3 ± 0.1	21 ± 3	6 ± 1
**202**	0.2 ± 0.1	21 ± 3	7 ± 1

Supplementary table 2

Fitted parameters for populations from figure 6. The ±-sign indicates the standard deviation of the populations (n > 35).

NRL (base pairs)	*N*	*k* (pN/nm)	ΔG_1 (k_BT)	ΔG_2 (k_BT)
**167 WT**	14	0.9 ± 0.1	24 ± 1	5 ± 1
**167 gH4**	14	0.3 ± 0.1	15 ± 1	5 ± 1
**197 WT**	10	0.3 ± 0.1	21 ± 1	6 ± 1
**197 gH4**	11	0.2 ± 0.1	20 ± 1	6 ± 1

Supplementary table 3

Fit parameters for chromatin fibers in figure 7a. The ±-sign indicates the standard error of fit.

NRL (base pairs)	histones	*k* (pN/nm)	ΔG_1 (k_BT)	ΔG_2 (k_BT)
**167**	*Hs* WT	1.3 ± 0.5	24 ± 3	7 ± 1
**167**	*Dm* WT	0.8 ± 0.4	22 ± 2	6 ± 1
**167**	*Dm* gH4	0.5 ± 0.2	17 ± 2	6 ± 1
**197**	*Hs* WT	0.3 ± 0.1	23 ± 2	7 ± 1
**197**	*Dm* WT	0.2 ± 0.1	20 ± 2	6 ± 1
**197**	*Dm* gH4	0.3 ± 0.1	20 ± 2	6 ± 1

Supplementary table 4

Population parameters for figure 7c-e. The ±-sign indicates the standard deviation (n > 42).

NRL (base pairs)	N_8	N_4	k (pN/nm)	ΔG_1 (k_BT)	ΔG_2 (k_BT)
**167**	15	0	1.1 ± 0.1	23 ± 1	9 ± 1
**168**	20	6	0.4 ± 0.1	17 ± 1	6 ± 1
**169**	18	3	0.4 ± 0.1	17 ± 1	6 ± 1
**170**	17	0	0.5 ± 0.1	19 ± 1	8 ± 1
**171**	19	6	0.5 ± 0.1	19 ± 1	8 ± 1
**172**	16	2	0.4 ± 0.1	19 ± 1	8 ± 1
**173**	21	6	0.4 ± 0.1	20 ± 1	8 ± 1
**174**	19	1	0.5 ± 0.1	20 ± 1	8 ± 1
**175**	20	1	0.4 ± 0.1	20 ± 1	9 ± 1
**176**	17	3	0.8 ± 0.1	26 ± 1	7 ± 1
**177**	17	1	0.8 ± 0.1	27 ± 1	8 ± 1
**192**	17	0	0.2 ± 0.1	16 ± 1	8 ± 1
**193**	18	4	0.2 ± 0.1	19 ± 1	8 ± 1
**194**	12	0	0.3 ± 0.1	21 ± 1	9 ± 1
**195**	16	5	0.3 ± 0.1	21 ± 1	7 ± 1
**196**	19	7	0.4 ± 0.1	22 ± 1	7 ± 1
**197**	18	0	0.3 ± 0.1	23 ± 1	8 ± 1
**198**	17	6	0.5 ± 0.1	23 ± 1	7 ± 1
**199**	16	3	0.3 ± 0.1	24 ± 1	8 ± 1
**200**	16	1	0.3 ± 0.1	22 ± 1	9 ± 1
**201**	15	4	0.2 ± 0.1	21 ± 1	7 ± 1
**202**	12	0	0.2 ± 0.1	20 ± 1	8 ± 1

Supplementary table 5

Fitted parameters for chromatin fibers in figure S1. The ±-sign indicates the standard error of fit.

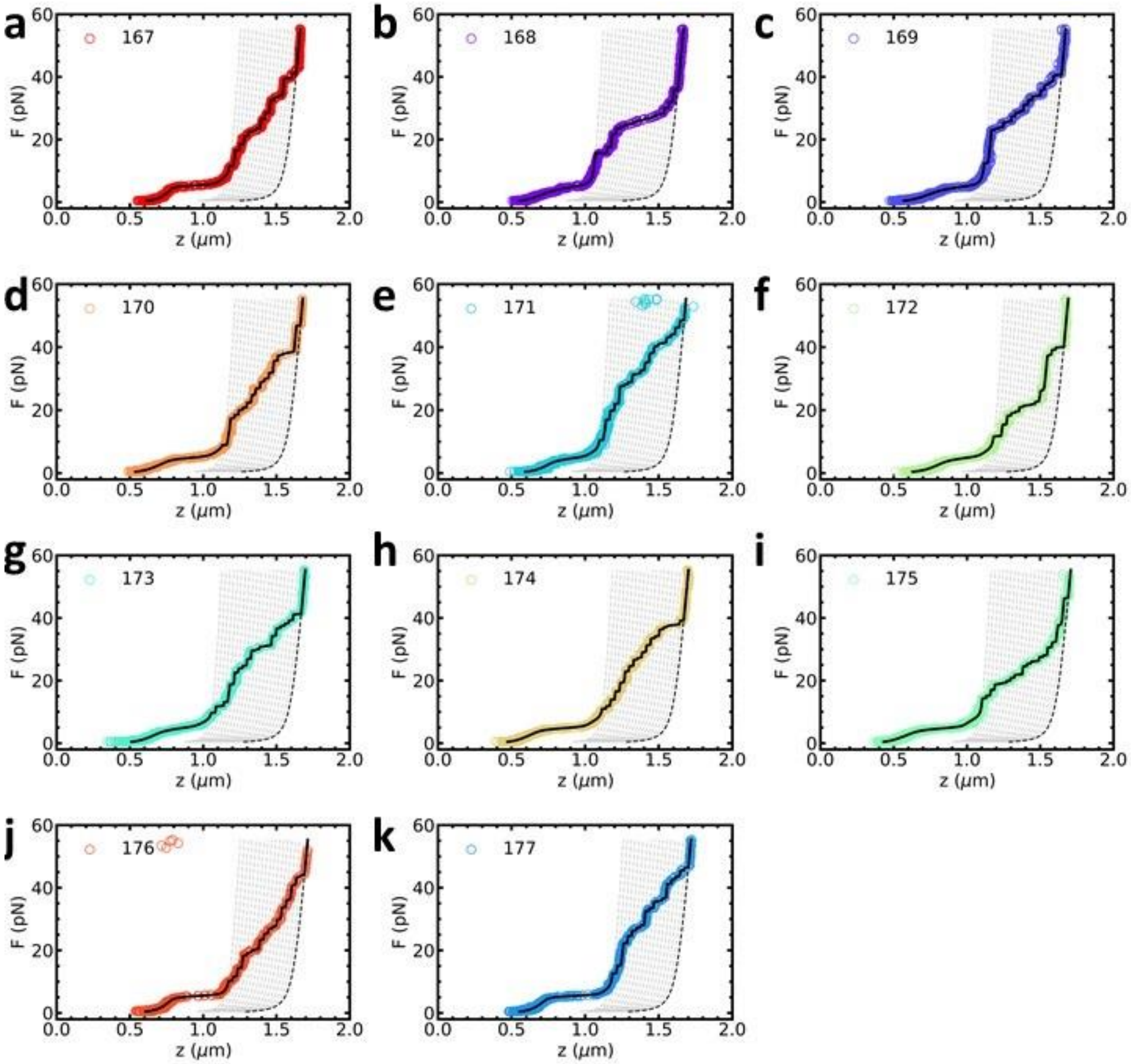

Supplementary figure 1

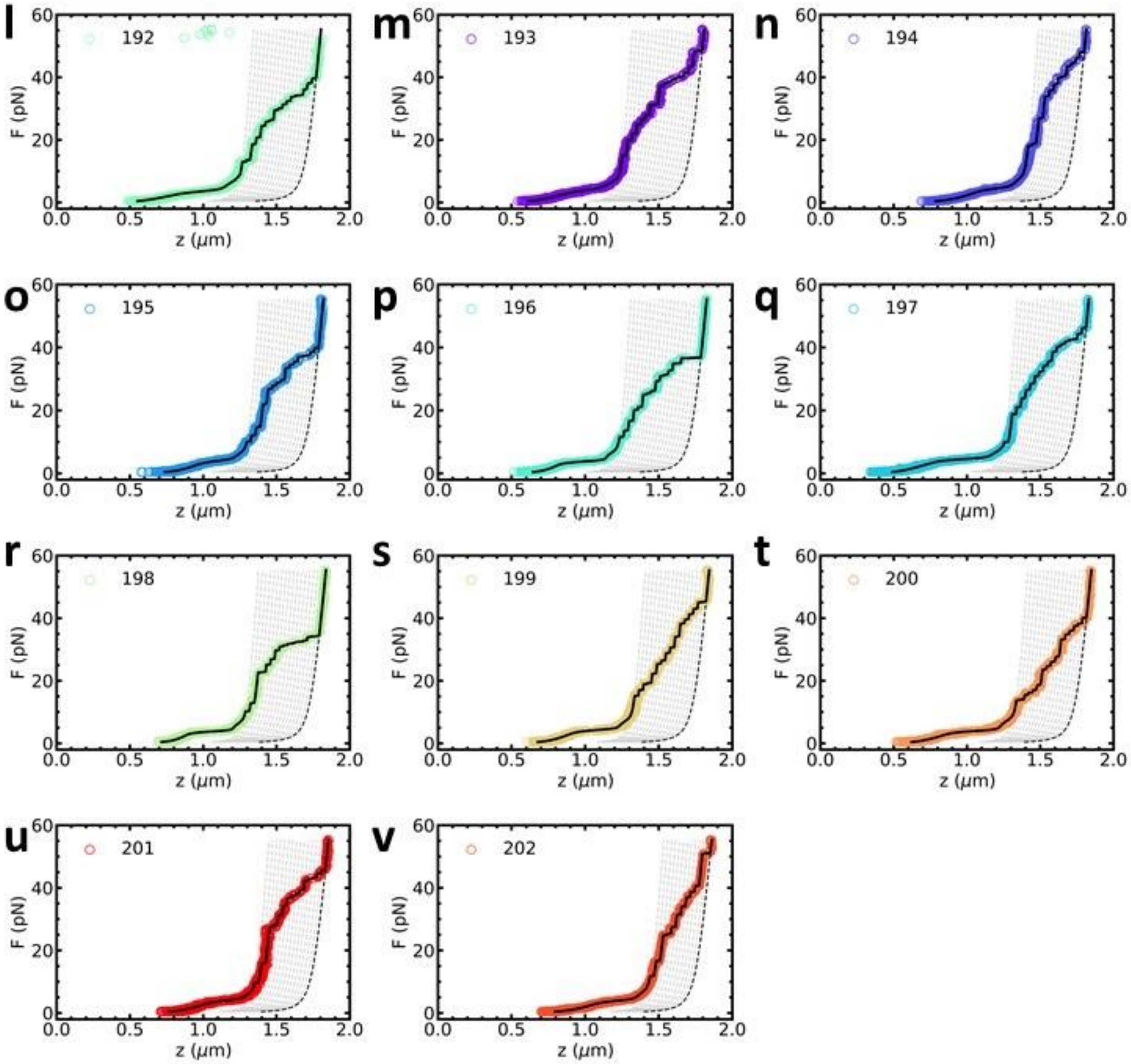

Supplementary figure 1, continued

Typical force-extension curves show unique chromatin fiber unfolding features for different NRLs. In general, all fibers unfolded at low forces ($f < 5$ pN). The fibers featured a small second transition at slightly higher forces ($f \approx 7$ pN) and stepwise unwrapping at high forces ($f > 10$ pN). Unwrapping intermediate states are indicated by the grey dashed lines. When all nucleosomes were completely unwrapped, the data followed the WLC, indicated by the black dashed line. Curves were depicted in range NRL 167 – 177 (a-k) and NRL 192 – 202 (l-v). Only the pulling trace is shown. The fitted parameters for each curve were given in table 5 of the supplementary material. Although some tethers ruptured at high forces (*e.g.* NRL 171, 176 and 192), the curve could still be fitted and used for statistics.

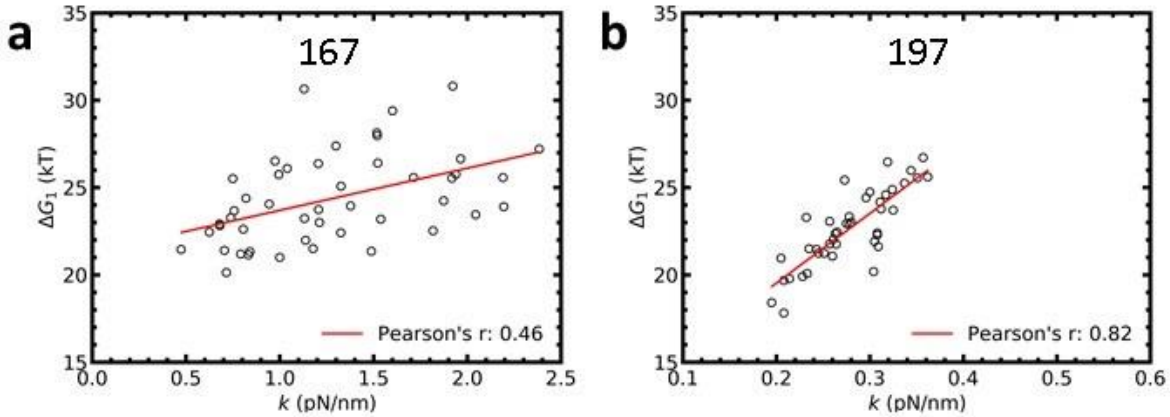

Supplementary figure 2

The correlation between stiffness k and rupture energy ΔG_1 hints at local defects in the fiber. a) Chromatin fibers with NRL 167 featured a relatively low correlation between k and ΔG_1 ($R \sim 0.5$). b) The correlation was stronger for NRL 197 ($R \sim 0.8$). Such increased correlation could point to defects in the composition of or nucleosome stacking in the fiber, which partially invalidate the statistical mechanics model, that assumes that all nucleosomes feature the same stages of unfolding.

References

1. de Jong BE, Brouwer TB, Kaczmarczyk A, et al (2018) Rigid Basepair Monte Carlo Simulations of One-Start and Two-Start Chromatin Fiber Unfolding by Force. Biophys J 115:1848–1859. https://doi.org/10.1016/J.BPJ.2018.10.007

2. Lowary P. T, Widom J (1998) New DNA sequence rules for high affinity binding to histone octamer and sequence-directed nucleosome positioning. J Mol Biol 276:19–42. https://doi.org/10.1006/jmbi.1997.1494

3. Brouwer TB, Kaczmarczyk A, Pham C, van Noort J (2018) Unraveling DNA Organization with Single-Molecule Force Spectroscopy Using Magnetic Tweezers. Humana Press, New York, NY, pp 317–349

4. Te Velthuis AJWW, Kerssemakers JWJJ, Lipfert J, Dekker NH (2010) Quantitative guidelines for force calibration through spectral analysis of magnetic tweezers data. Biophys J 99:1292–1302. https://doi.org/10.1016/j.bpj.2010.06.008

5. Vilfan ID, Lipfert J, Koster DA, et al (2009) Magnetic Tweezers for Single-Molecule Experiments. In: single-molecule biophysics. pp 371–395

6. Meng H, Andresen K, van Noort J (2015) Quantitative analysis of single-molecule force spectroscopy on folded chromatin fibers. Nucleic Acids Res 43:3578–3590. https://doi.org/10.1093/nar/gkv215

7. Tinoco Jr I, Bustamante C (2002) The effect of force on thermodynamics and kinetics of single molecule reactions. Biophys Chem 101–102:513–533. https://doi.org/10.1016/S0301-4622(02)00177-1

8. Meng H, Andresen K, van Noort J (2015) Quantitative analysis of single-molecule force spectroscopy on folded chromatin fibers. Nucleic Acids Res 43:3578–3590. https://doi.org/10.1093/nar/gkv215

9. Bustamante C, Bryant Z, Smith SB (2003) Ten years of tension: single-molecule DNA mechanics. Nature 421:423–427. https://doi.org/10.1038/nature01405

10. Strick T, Allemand J-F, Croquette V, Bensimon D (2000) Twisting and stretching single DNA molecules. Prog Biophys Mol Biol 74:115–140. https://doi.org/10.1016/S0079-6107(00)00018-3

11. Korolev N, Nordenskiöld L, Lyubartsev AP (2016) Multiscale coarse-grained modelling of chromatin components: DNA and the nucleosome. Adv Colloid Interface Sci 1–13. https://doi.org/10.1016/j.cis.2016.02.002

12. Kaczmarczyk A (2019) Nucleosome stacking in chromatin fibers: probed with single-molecule force- and torque-spectroscopy. Leiden University

13. Korolev N, Lyubartsev AP, Nordenskiöld L (2018) A systematic analysis of nucleosome core particle and nucleosome-nucleosome stacking structure. Sci Rep 8:1543. https://doi.org/10.1038/s41598-018-19875-0
